# Twisting Right to Left: A…A Mismatch in a CAG Trinucleotide Repeat Overexpansion Provokes Left-Handed Z-DNA Conformation

**DOI:** 10.1371/journal.pcbi.1004162

**Published:** 2015-04-13

**Authors:** Noorain Khan, Narendar Kolimi, Thenmalarchelvi Rathinavelan

**Affiliations:** Department of Biotechnology, Indian Institute of Technology Hyderabad, Kandi, Telangana State, India; Baltimore, UNITED STATES

## Abstract

Conformational polymorphism of DNA is a major causative factor behind several incurable trinucleotide repeat expansion disorders that arise from overexpansion of trinucleotide repeats located in coding/non-coding regions of specific genes. Hairpin DNA structures that are formed due to overexpansion of CAG repeat lead to Huntington’s disorder and spinocerebellar ataxias. Nonetheless, DNA hairpin stem structure that generally embraces B-form with canonical base pairs is poorly understood in the context of periodic noncanonical A…A mismatch as found in CAG repeat overexpansion. Molecular dynamics simulations on DNA hairpin stems containing A…A mismatches in a CAG repeat overexpansion show that A…A dictates local Z-form irrespective of starting *glycosyl* conformation, in sharp contrast to canonical DNA duplex. Transition from B-to-Z is due to the mechanistic effect that originates from its pronounced nonisostericity with flanking canonical base pairs facilitated by base extrusion, backbone and/or base flipping. Based on these structural insights we envisage that such an unusual DNA structure of the CAG hairpin stem may have a role in disease pathogenesis. As this is the first study that delineates the influence of a single A…A mismatch in reversing DNA helicity, it would further have an impact on understanding DNA mismatch repair.

## Author Summary

When a set of 3 nucleotides in a DNA sequence repeats beyond a certain number, it leads to incurable neurological or neuromuscular disorders. Such DNA sequences tend to form unusual DNA structures comprising of base pairing schemes different from the canonical A…T/G…C base pairs. Influence of such unusual base pairing on the overall 3-dimensional structure of DNA and its impact on the pathogenesis of disorder is not well understood. CAG repeat overexpansion that leads to Huntington’s disorder and several spinocerebellar ataxias forms noncanonical A…A base pair in between canonical C…G and G…C base pairs. However, no detailed structural information is available on the influence of an A…A mismatch on a DNA structure under any sequence context. Here, we have shown for the first time that A…A base pairing in a CAG repeat provokes the formation of left-handed Z-DNA due to the pronounced structural dissimilarity of A…A base pair with G…C base pair, leading to periodic B-Z junction. Thus, these results suggest that formation of periodic B-Z junction may be one of the molecular bases for CAG repeat instability.

## Introduction

Apart from the ‘canonical’ B-DNA conformation, DNA can also adopt a variety of ‘non-canonical’ conformations such as hairpin, triplex and tetraplex depending on the sequence and environment. It is well known that formation of such unusual non-B-DNA structures during the overexpansion of trinucleotide microsatellites (tandem repeats of 1–3 nucleotide length) is responsible for at least 22 incurable trinucleotide repeat expansion disorders (TREDs) that are mainly neurological or neuromuscular in nature[1,2,3,4,5]. For instance, occurrence of hairpin structure due to the abnormal increase in the CTG repeat length in the untranslated region of DMPK gene causes myotonic dystrophy type-1[6,7]. Likewise, hairpin formation in CAG repeat expansion located in the protein-coding region leads to Huntington’s disorder & several spinocerebellar ataxias[7]. Direct evidence for the role of such hairpin structure in instigating replication-dependent instability has been demonstrated for the first time in human cells with 5’CTG.5’CAG microsatellite overexpanion[8]. Recently, it has been shown that CAG repeat overexpansion in DNA leads to toxicity by triggering cell death[9,10] and thus, warranting a detailed investigation on the hairpin structures formed under such abnormal expansion.

Although diverse mechanisms at DNA, RNA and protein levels have been identified for the progression of TREDs[11], until now, the main focus as potential therapeutic targets has been on RNA and protein levels. In fact, crystal structures of RNA duplex (hairpin stems) containing CUG[12] and CAG[13] repeats that form noncanonical U…U[12] & A…A[13] base-pairs offers useful information as the pathogenic CUG and CAG RNA hairpins have a role in misregulating the alternative splicing by MBNL1[14], leading to neurotoxicity. Though the isosequential DNA also intends to form hairpin structure[15], detailed structural insights about DNA duplex with CAG and CTG repeats that form A…A and T…T mismatches respectively are still inaccessible. With emerging evidence on ‘DNA toxicity’ of CAG repeat overexpansion[9,10], such structural information would facilitate the understanding of underlying mechanisms behind repeat instability at DNA level which is yet another potential drug target. In this context, we aim here to investigate the structure and dynamics of DNA duplex containing CAG repeat using molecular dynamics (MD) simulation technique. Surprisingly, results of the MD simulations indicate that A…A mismatch in a CAG repeat overexpansion induces periodic B-Z junction irrespective of the starting conformation. Thus, we suggest that such an unusual DNA structure of CAG hairpin stem may affect the biological function and may be one of the factors responsible for ‘DNA toxicity’ [9,10].

## Results

### Non-canonical A…A mismatch induces Z-DNA sandwich structure

**Fig 1:**
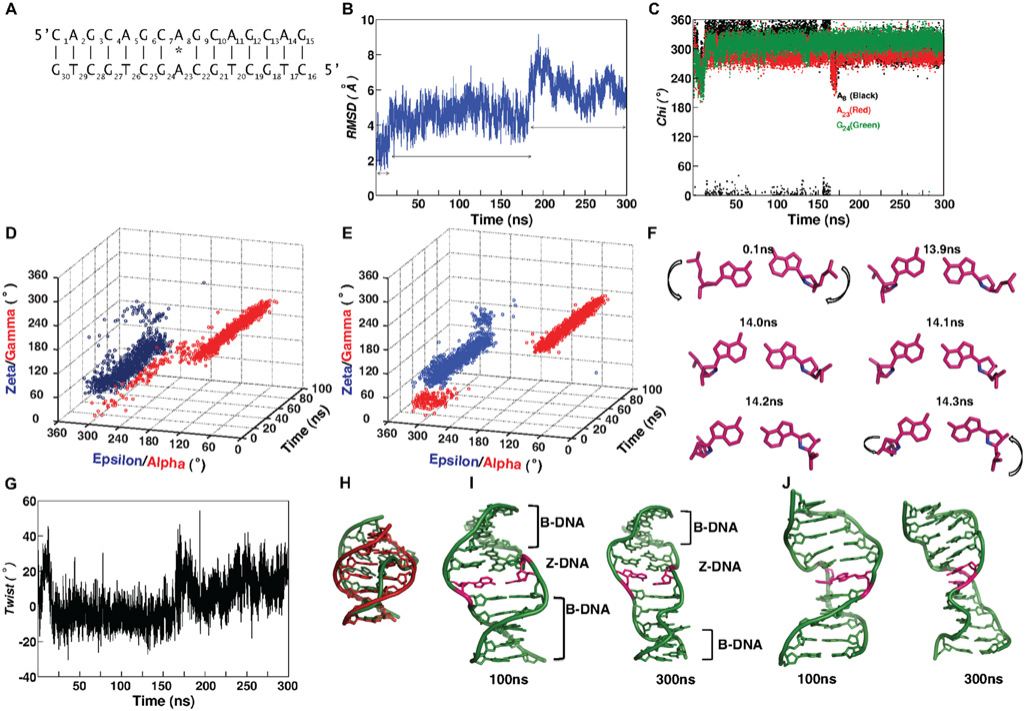
Sugar-phosphate flipping mediated B- to Z-DNA conformational transition. (A) 15mer DNA duplex with a single A_8_…A_23_ mismatch used for MD simulation. (B) Time vs RMSD and (C) Time vs *chi* profiles over 300ns simulation. (D) 3D plot showing the relationship between alpha & gamma and epsilon & zeta over 100ns simulation at the A_8_G_9_ and (E) G_24_C_25_ steps. (F) Flipping of sugar-phosphate (marked by arrow) backbone during 13.9–14.3ns at A_8_…A_23_ mismatch site. For comparison A_8_…A_23_ mismatch at 0.1ns is shown. O4’ atom of the sugar is colored blue. (G) Time vs twist profile at C_7_A_8_ step over 300ns simulation. (H) Superposition of 0.1ns (red) and 15ns (green) average structures (central 7mer). Note the effect of negative twist in forming Z-DNA like structure. (I, J) Average structure calculated over 99.9-100ns and 299.9-300ns (central 11mer) showing the Z-DNA sandwich (Z-DNA flanked by B-DNA): minor (I) and (J) major grooves at the mismatch (colored pink) site facing the viewer. Associated expansion in the minor groove and increase in Z-DNA stretch with respect to time can be seen in I.

Role of a single noncanonical A_8_…A_23_ pair amidst canonical base pairs (Fig. 1A) is investigated through 300ns MD simulation, prior to the investigation of CAG repeats with periodic A…A mismatch as in Huntington’s disorder. As CAG repeat containing RNA crystal structures[13]_,_[16] exhibit two different *glycosyl* conformations for A…A mismatch, 2 starting models with N6(A_23_)…N1(A_8_) hydrogen bond are considered for MD simulation: one with *anti…anti* (~250°) and the other with +*syn*(~79°)*…anti*(~250°) base *glycosyl* conformation.

### A…A disfavors *anti…anti glycosyl* conformation

Root mean square deviation (RMSD) calculated over 300ns simulation indicates the existence of three different ensembles (Fig. 1B): the first ensemble persists till ~16.5ns with RMSD centered around 2.8(0.7)Å, the second one persists between 16.5-181ns with a RMSD of 4.7(0.7)Å and the third one persists beyond ~181ns with the highest RMSD of 6.2(0.8)Å.

Intriguingly, a high RMSD of 4.5(0.6)Å observed between 16.5-100ns is associated with a change in *glycosyl* conformation of mismatched A_23_ and A_8_ from the starting *anti* conformation to -*syn* conformation.

During the first 16.5ns, A_8_ and A_23_ fluctuate between -*syn* and *anti glycosyl* conformations. Beyond 16.5ns, both A_8_ [-38(17)] and A_23_ [-66(17)] stay in -*syn* conformation (Fig. 1C). Similar tendency is also seen in the neighboring G_24_, wherein, it prefers -*syn* [309 (15°)] conformation beyond 16.5ns (Fig. 1C). Thus, it is clear that A_8_…A_23_ mismatch disfavors *anti…anti glycosyl* conformation and causes distortion in the duplex.

Aforementioned conformational changes in *chi* are accompanied by transformations in sugar-phosphate backbone at and around the mismatch site. For instance, during the first 100ns simulation, A_8_G_9_&G_24_C_25_ steps exhibit the characteristics of Z-DNA. The conformational angles (ε,ζ,α,γ) at A_8_G_9_ favor (*g* ^*-*^,*g* ^*+*^,*g* ^*+*^,*trans*) [283(11°), 83(14°), 99(48°), 181(36°)] (Fig. 1D). Similar tendency is seen at G_24_C_25_ step with (ε,ζ,α,γ) favoring (293(15°), 89(13°), 79(14°), 199(13°)) conformation (Fig. 1E). These conformational rearrangements lead to transformation from right-handed B to left-handed Z form at the A_8_…A_23_ mismatch site leading to the formation of B-Z junction. These changes happen mainly due to the sugar phosphate flipping (S1–S3 Movie), which can clearly be seen from the repositioning of O4’ atoms (Fig. 1F, colored blue) of A_8_&A_23_ sugars as well as the sugar-phosphate backbone (Fig. 1F, indicated in arrow).

Strikingly, the effect of left-handed Z-DNA conformation observed between 16.5-100ns is also reflected in the helical twist angle of C_7_A_8_.A_23_G_24_ step which favor low (negative) twist of −4° (7) (Fig. 1G) flanked by high (positive) twists at the neighboring G_6_C_7_ (32 (4°)) & A_8_G_9_ (31(6°)) steps (S1 Fig). These, together with the conformational changes at A_23_…A_8_ mismatch reflect in the helicity of the duplex, which can be clearly seen from the superposition of average structures calculated over 1-100ps and 14.9-15ns (Fig. 1H). While the former is in B-form conformation, the latter shows a change in helicity leading to local Z-DNA formation. Occurrence of a low negative twist due to local Z-DNA formation in the midst of high twists at G_6_C_7_A_8_G_9_ stretch leads to local unwinding of the helix as can be seen Fig. 1I. As A_23_…A_8_ mismatch site is located exactly in the middle of DNA (Fig. 1A), aforementioned distortions lead to Z-DNA sandwich, viz., a mini Z-DNA is embedded in a B-DNA. Essentially, similar features are observed in B-Z junction formed by L-deoxy guanine and L-deoxy cytosine (S1 Fig).

As the Z-DNA formation happens due to the sugar-phosphate flipping, hydrogen bond between A_8_&A_23_ undergoes minor changes (S2 Fig). During the first ~16.5ns, N1(A_8_)…N6(A_23_) hydrogen bond persists, whereas, between 16.5-100ns, N1(A_23_)…N6(A_8_) hydrogen bond is predominantly favored due to the slight movement of A_23_ towards the minor groove. Base extrusion at the mismatch site is also observed during 100ns simulation.

**Fig 2:**
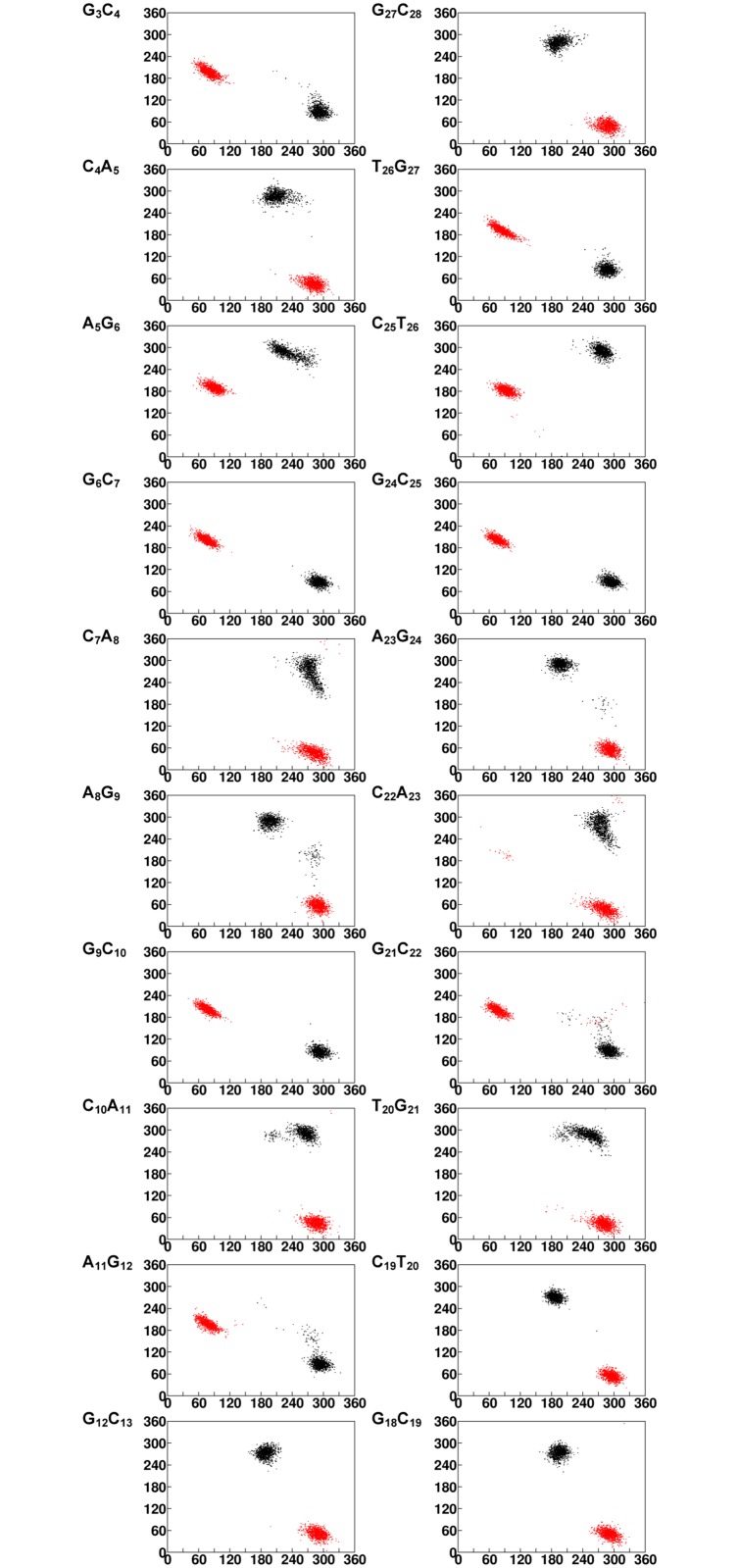
2D plots indicating backbone conformational preference for d(CAG)_2_CAG(CAG)_2_.d(CTG)_2_CAG(CTG)_2_ duplex with A_8_…A_23_ in *anti…anti* starting *glycosyl* conformation during the last 10ns. (ε&ζ) and (α&γ) 2D plots corresponding to the first strand is given in 2^nd^ column along with the appropriate step marked in the 1^st^ column. (ε&ζ) and (α&γ) 2D plots corresponding to the second strand is given in 4^th^ column along with the appropriate step marked in the 3^rd^ column. 2D plots of (ε&ζ) and (α&γ) are marked in black & red respectively. Note that ε&α are represented in X-axis and ζ&γ are represented in Y-axis.

B-Z junction induced by A_8_…A_23_ mismatch propagates to the neighboring bases (A_5_ to A_11_) beyond 181ns (Fig. 1I-J), which reflects in the highest RMSD of 6.2Å (Fig. 1B). Though the *chi* angle at A_8_, A_23_ and G_24_ remain in -*syn* conformation (Fig. 1C) as the 1^st^ 100ns simulation (see above), (ε,ζ,α,γ) at the A_8_G_9_ step takes up (*trans*,*g* ^*-*^,*g* ^*-*^,*g* ^*+*^) with C_7_A_8_.A_23_G_24_ step adopting a slightly higher helical twist of 11.1(9°) (Fig. 1G). However, (ε,ζ,α,γ) at G_3_C_4_, G_6_C_7_, G_9_C_10_, A_11_G_12_, G_21_C_22_, G_24_C_25_ and T_26_G_27_ step also favor (*g* ^*-*^,*g* ^*+*^,*g* ^*+*^,*trans*) (Fig. 2). Additionally, A_5_G_6_&C_25_T_26_ favor (*g* ^*-*^,*g* ^*-*^,*g* ^*+*^,*t*) for (ε,ζ,α,γ), while C_7_A_8_ takes up (*g* ^*-*^,*g* ^*-*^,*g* ^*-*^,*g* ^*+*^). This eventually reflects in the helical twist angle at the central A_5_G_6_, G_6_C_7_, C_7_A_8_, A_8_G_9_ & C_10_A_11_ adopting lower helical twist (S1 Fig). Notably, (*g* ^*-*^,*g* ^*-*^,*g* ^*+*^,*t*) conformation for A_5_G_6_ and for its complementary C_25_T_26_ step results in a negative twist of -10°. Thus, there is an evident increase in Z-DNA stretch at & around the mismatch site during the end of the simulation. It is noteworthy that beyond 181ns, N1(A_8_)…N6(A_23_) & N1(A_23_)…N6(A_8_) hydrogen bonds are equally favorable, while the canonical C_7_…G_24_ and G_9_…C_22_ hydrogen bonds flanking the A_8_…A_23_ mismatch remain unaffected throughout the simulation (S2 Fig).

Concomitant to above, major and minor groove widths also undergo changes. Unwinding of the helix leads to the expansion of minor groove width at the mismatch site to ~20.1(0.4)Å flanked by comparatively narrower groove widths of 12.7Å&14.9Å on either side at the end of the 300ns simulation.

### A…A disfavors *+syn…anti* glycosyl conformation

**Fig 3:**
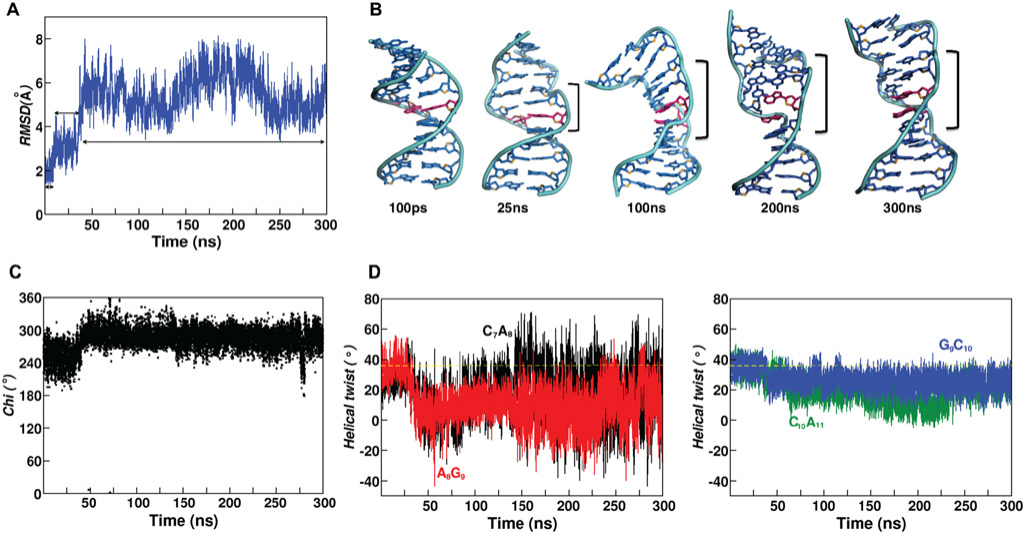
Z-DNA sandwich structure formed by A_8_…A_23_ mismatch with *+syn…anti* starting *glycosyl* conformation for sequence given in Fig. 1A. (A) Time vs RMSD profile showing three different ensembles during the 300ns simulation. (B) Cartoon diagram of representative average structures (calculated over 100ps) corresponding to the 4 different time intervals. Note the increase in the Z-DNA stretch (marked by square bracket) with respect to time. (C) 2D plot showing the conformational transformation occurring in *chi* at A_8_. (D) Time vs helical twist profile showing the preference for low twist at C_7_A_8_, A_8_G_9_, G_9_C_10_ and C_10_A_11_ steps due to the local Z-DNA formation.

Akin to A_8_…A_23_ mismatch with *anti…anti glycosyl* starting conformation, the starting model with *+syn*…*anti glycosyl* conformation also undergoes significant conformational changes. This can be seen from RMSD (Fig. 3A) that increases to 2(0.3)Å till ~9.1ns and subsequently to 3.1(0.5)Å during 9.1-36ns. It stays ~5.5(0.8)Å beyond 36ns.

Detailed analysis indicates that the increase in RMSD to 5.5Å is due to the conformational preference for local Z-DNA structure at & around the A_8_…A_23_ mismatch site to accommodate the mismatch. In fact, an increase in Z-DNA stretch around the mismatch site is seen (Fig. 3B) during the 300ns simulation. One of the marked changes associated with Z-DNA conformational preference is A_8_ adopting *high-anti/-syn* (287 (17°)) *glycosyl* conformation beyond 36ns (Fig. 3C). Conformational changes at A_8_ beyond 36ns enforce -*syn glycosyl* conformation for neighboring G_9_ (248 (26°) to 321(32°)) and G_24_ (248(25°) to 324 (15°)) (S3A Fig). Other notable changes that happen during the early part of the simulation (~9ns) in seeding Z-DNA conformation are, the preference for -*syn glycosyl* conformation by G_21_ (from 249(24°) to 296(25°)) (hydrogen bonded with C_10_) and A_11_ (from 257(23°) to 302(32°)) (base paired with T_20_) that are located in the neighborhood of A_8_…A_23_ mismatch site (S3A Fig). Irrespective of the above conformational changes, *chi* at A_23_ stays close to the initial *+syn* (S3A Fig) conformation throughout the simulation. It is noteworthy that a total loss of hydrogen bonds at N1(A_8_)…N6(A_23_) & N6(A_8_)…N7(A_23_) that happenes due to base extrusion during 30-40ns facilitates B-Z transition (S3B-E Fig).

**Fig 4:**
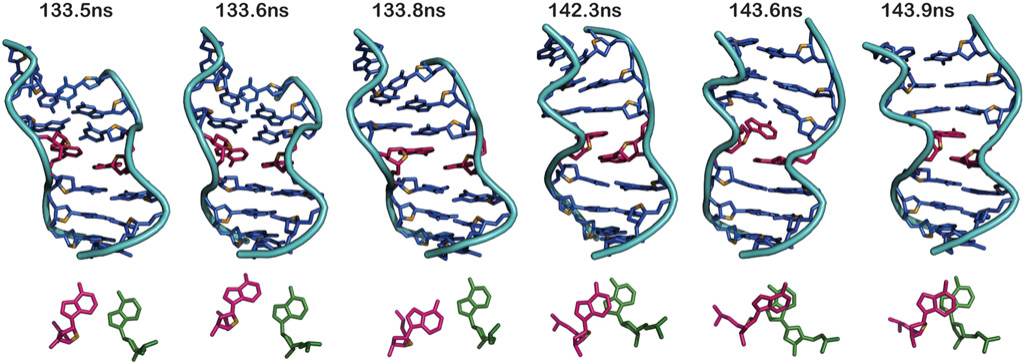
Z-DNA sandwich structure formed by A_8_…A_23_ mismatch with *+syn…anti* starting *glycosyl* conformation (Fig. 1A) promotes intercalation between the mismatched bases. (Top) Snapshots of central 7mer illustrating the formation of intercalated A_8_&A_23_ (colored magenta) during 133-144ns and (Bottom) the associated interaction between A_8_ (magenta) & A_23_ (green). Note the loss of hydrogen bond at 133.6ns following which, A_8_&A_23_ move out of plane with each other (133.8ns). Subsequently, A_8_ stacks onto A_23_ completely ~142ns and stays in the same conformation till the end of the simulation.

Yet another interesting observation is the preference for stacked conformation between the mismatched A_8_&A_23_ bases (Fig. 4) that is facilitated by the Z-DNA conformation. As a result, there is a total loss of N1(A_8_)…N6(A_23_) hydrogen bond as well as N6(A_8_)…N7(A_23_) hydrogen bond between the mismatched bases beyond 150ns (S3B Fig). It happens in such a way that ~133ns the hydrogen bond becomes longish, followed by A_8_ and A_23_ moving out-of-plane with each other. Subsequently, A_8_ stacks on top of A_23_ like an intercalator and stays till the end of the simulation (Fig. 4). During the aforementioned conformational changes, the canonical C_7_…G_24_ and G_9_…C_22_ that is located above and below the A_8_…A_23_ mismatch respectively remain intact (S3B Fig).

Excitingly, aforementioned transformations are accompanied by prevalence for Z-DNA backbone conformation. For instance, when both A_8_ & A_23_ are in plane during the first 100ns simulation, -*syn* conformation for *chi* at A_8_, G_9_, G_21_, G_24_ & A_11_ is concomitant with (ε,ζ,α,γ) adopting (*g* ^*-*^,*g* ^*+*^,*g* ^*+*^,*trans*) at G_9_C_10_ (S4A Fig), A_11_G_12_ (S4B Fig) & G_24_C_25_ (S4F Fig) steps, while T_20_G_21_ (S4C Fig), C_22_A_23_ (S4D Fig) & A_23_G_24_ (S4E Fig) steps taking up (*g* ^*-*^,*g* ^*-*^,*g* ^*+*^,*trans*). Consequent to the above sugar-phosphate conformational changes, helical twists at C_7_A_8_ (8.5 (12))°, A_8_G_9_ (7.4 (8))° and C_10_A_11_ (6.1 (9))° (Fig. 3D) steps adopt low twist values in between high twist values (S5B Fig) resulting in a Z-DNA sandwich structure as before.

Stacked conformation of A_8_&A_23_ that is formed after 150ns leads to large fluctuation in the helical twist of C_7_A_8_ & A_8_G_9_ steps, wherein, the C1’…C1’ vector of A_8_…A_23_ is nearly perpendicular to the C1’…C1’ vectors of the neighboring canonical base pairs. This is associated with large fluctuation in conformational angle alpha at C_22_A_23_ step (S6 Fig). Additionally, (ε,ζ,α,γ) for G_9_C_10_, A_11_G_12_, G_21_C_22_, G_24_C_25_, C_10_A_11_ & T_20_G_21_ steps also favor Z-DNA conformations like (*g* ^*-*^,*g* ^*+*^,*g* ^*+*^,*trans*) & (*g* ^*-*^,*g* ^*-*^,*g* ^*-*^,*trans*) (S6 Fig). The general tendency in helical twist associated with the above conformational preference is that A_8_G_9_ (8 (11))°, G_9_C_10_ (25(5))° and C_10_A_11_ (18 (8))° prefer a low twist during the 150-300ns (S5C,D Fig).

It is clear from above that like in the previous situation (Fig. 1), formation of local Z-DNA conformation is propagated to the neighboring bases (from C_7_ to G_12_) of A_8_…A_23_ mismatch. This eventually reflects in at least 3 steps located in the middle of the duplex taking up lower helical twists (S5B-D Fig). Essentially, this leads to unwinding of the double helix (S5B-D Fig & S7 Fig and S4 Movie), a typical characteristic of B-Z junction (PDB ID: 1FV7). Such unwinding is accompanied by expansion in the major (maximum of 28 Å) and minor (maximum of 20 Å) groove widths. However, at the mismatch site, the minor groove width shrinks to 11.5 Å during the 100ns simulation. It further shrinks to 8 Å, followed by the stacked conformation of A_8_&A_23_.

Thus, formation of a local Z-DNA conformation accompanied by unwinding of the helix is evident even with a single A…A mismatch irrespective of the starting conformation.

### Periodic B-Z junction in (CAG)_6_. (CAG)_6_ duplex

**Fig 5:**
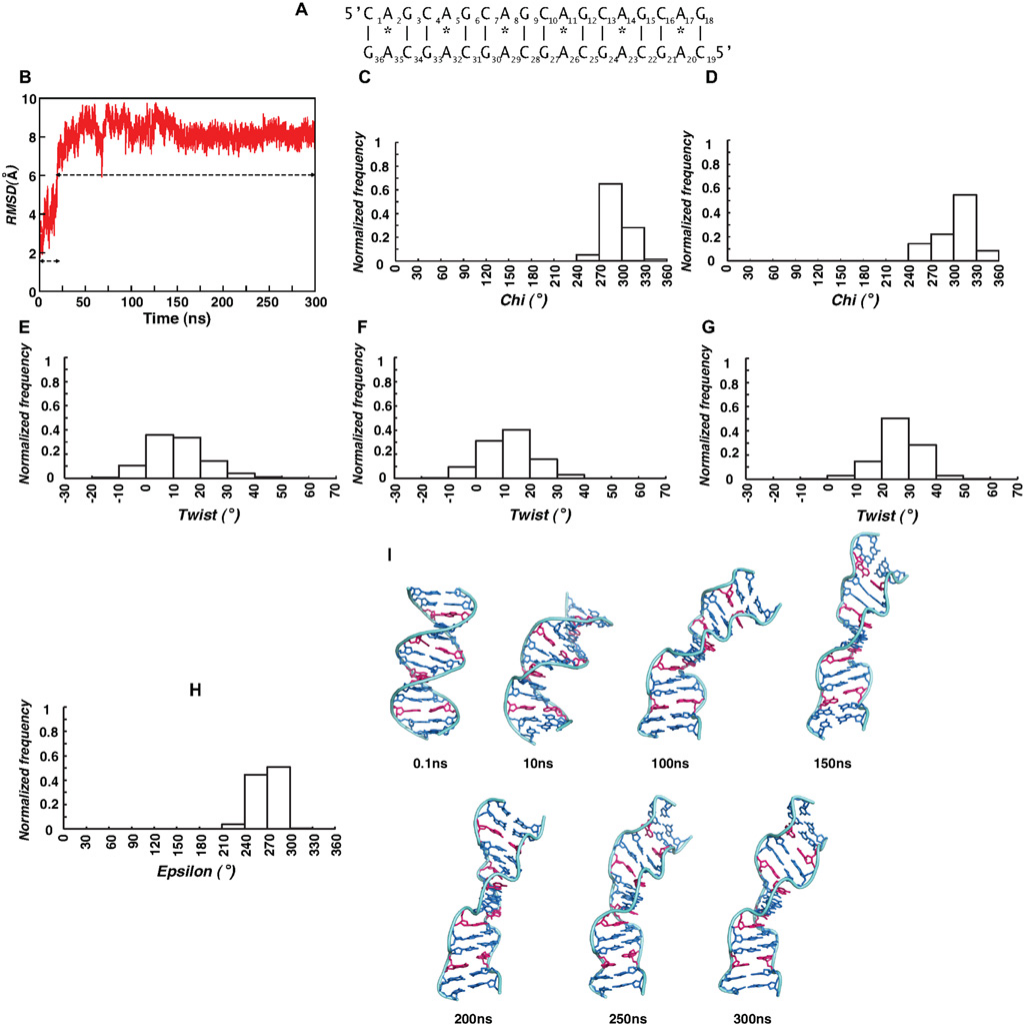
Periodic B-Z junction induced by recurring A…A mismatches in CAG repeat expansion. (A) 18mer DNA duplex with 6 A…A mismatches used in the present MD simulation. (B) Time vs RMSD profile showing the significant conformational change from the starting model. (C-H) Histogram corresponding to: *glycosyl* conformation of (C) A’s & (D) G’s, twist angles at all the (E) CA (F) AG & (G) GC steps and epsilon at (H) CA step over 291-300ns. (I) Cartoon diagram showing the conformational changes from B- to Z-DNA during the simulation. Note that terminal 2 base pairs on either ends are not included due to end fraying effect.

To investigate the effect of periodic occurrence of A…A mismatch as in the real situation of Huntington’s disorder and several spinocerebellar ataxias, 300ns MD simulation has been carried out for d(CAG)_6_.d(CAG)_6_ sequence (Fig. 5A). As before, 2 starting models each with *+syn…anti* and *anti…anti glycosyl* conformations are considered for all the six A…A mismatches.

#### A…A pair with *anti…anti* starting conformation

A high RMSD of 8.2(0.5)Å beyond 25ns (Fig. 5B) implicates that the initial model with A…A mismatches in *anti…anti* starting conformation undergoes significant conformational rearrangement to accommodate the mismatches.

Such a high RMSD is associated with all the A’s (A_5_, A_8_, A_11_, A_14_, A_23_, A_26_, A_29_ and A_32_) preferring *high-anti*(65%) and -*syn*(28%) conformation (Fig. 5C). Intriguingly, G’s that flank A’s also have the preponderance for *high-anti* (22%) and -*syn* (55%) conformation (Fig. 5D), while the C’s retain *anti glycosyl* conformation. Concomitant to such *glycosyl* conformational change, sequence dependent twist angle variations are observed. The general tendency is that, while CA (11.7(10°)) (Fig. 5E) and AG (12(9°)) (Fig. 5F) steps adopt a low twist, the GC step favors a high twist (28(6°)) (Fig. 5G) resulting in periodic presence of a high & a low twist adjacent to each other, a characteristic similar to B-Z junction (S8 Fig).

Above conformational rearrangements are further concomitant with predominantly falling in *g* ^*-*^ (95%) conformation at the CA step (Fig. 5H). This eventually leads to (ε,ζ,α,γ) equally favoring (*g* ^*-*^,*g* ^*-*^,*g* ^*-*^,*g* ^*+*^) (BIII conformation wherein, (ε,ζ,α) adopts (*g* ^*-*^,*g* ^*-*^,*g* ^*-*^))[17] or (*g* ^*-*^,*g* ^*-*^,*g* ^*+*^,*trans*) at the CA step (S9(Top) Fig). Another notable observation is that 70% of ε at GC step favors *g* ^*-*^ conformation with γ invariably adopting *trans* conformation. Thus, GC step tends to prefer (*g* ^*-*^,*g* ^*+*^,*g* ^*+*^,*trans*) and (*t*,*g* ^*-*^,*g* ^*-*^,*t*) for (ε,ζ,α,γ) (S9(Bottom) Fig). However, AG step has the preponderance (greater than 80%) for BI, wherein, (ε,ζ,α) adopts (*t*,*g* ^*-*^,*g* ^*-*^) & BII, wherein, (ε,ζ,α) adopts (*g* ^*-*^,*t*,*g* ^*-*^) geometry (S9(Middle) Fig). Irrespective of these *glycosidic* and sugar-phosphate conformational changes, N1(A)…N6(A) hydrogen bond remains intact. Nonetheless, during the B- to-Z transition, base extrusion in A…A mismatch is also observed.

Aforementioned conformational changes caused by A…A mismatch at the CA and GC steps leads to sugar-phosphate backbone flipping causing helicity reversal that results in the formation of periodic B-Z junction (Fig. 5I). Formation of such B-Z junction also reflects in the solvation as both water and ion populate more in the minor groove than the major groove (S10 Fig).

Thus, it is clear that A…A pair in the midst of G…C&C…G pairs in a DNA duplex disfavors *anti…anti glycosyl* conformation and favors left-handed Z-form structure.

##### A…A pair with *+syn…anti* starting conformation

Inline with the above, 300ns MD simulation carried out for A…A mismatch with *+syn…anti glycosyl* starting conformation also reveals the preponderance for -*syn…-syn/ +syn…high-anti glycosyl* conformation. At the end of the simulation, out of 4 A…A mismatches, 2 of them adopt *+syn…high-anti* conformation (A_5_…A_32_&A_8_…A_29_), while the other 2 prefer -*syn…-syn* conformation (A_11_…A_26_&A_14_…A_23_).

**Fig 6:**
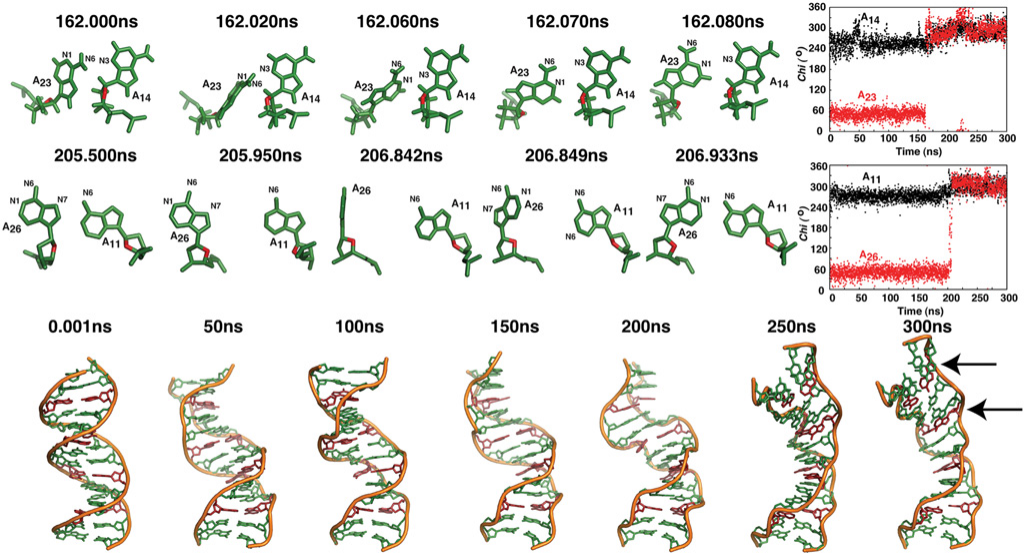
Local Z-DNA formation by ‘base flipping’ mechanism in CAG repeat with periodic occurrence of A…A mismatches. (Top) Snapshots of A_23_…A_14_ mismatch site illustrating base flipping of A_23_ from *+syn* to -*syn glycosyl* conformation through *cis glycosyl* conformation that occurs at ~162ns. Adoption of -*syn glycosyl* conformation by both A_14_ (black) & A_23_ (red) beyond 160ns can be seen in Time vs *chi* profile (Top row, Right most corner). (Middle) Snapshots of A_26_…A_11_ mismatch site indicating *+syn* to -*syn glycosyl* conformation of A_26_ through *trans glycosyl* conformation ~205ns. Both A_11_ (black) & A_26_ (red) assume -*syn glycosyl* conformation beyond 205ns as seen in Time vs *chi* profile (Middle row, Right most corner). (Bottom) Cartoon diagram illustrating the formation of local Z-DNA concomitant with the above mentioned base extrusion & base flipping (A…A mismatches colored maroon). Positions of A_23_…A_14_ and A_26_…A_11_ mismatches are indicated by arrow (Bottom row, Right most corner). Compact B-form structure at 0.001ns and the extended Z-DNA like structure at 300ns can be clearly visualized (Bottom). Note that in all the figures the time associated with each snapshot is indicated.

Most intriguingly, the transition to -*syn…-syn* takes place through base flipping (Fig. 6(top, middle), S5&S6 Movie). At A_14_…A_23_ base pair, ~80ns N3(A_14_)…N6(A_23_) hydrogen bond evolves instead of the initial N6(A_14_)…N1(A_23_) due to the movement of A_23_ towards the minor groove and stays till ~162ns. Just in 200ps (between 162–162.2ns), base flipping occurs accompanied by -*syn glycosyl* conformation for A_23_&A_14_ (Fig. 6(top), S11 Fig and S5 Movie). Similarly, at A_26_…A_11_ mismatch site, base extrusion happens ~205ns resulting in a total loss of hydrogen bond (Fig. 6(middle), S6 Movie). Soon after, A_26_ undergoes base flipping and establishes N6(A_26_)…N1(A_11_) hydrogen bond concomitant with -*syn glycosyl* conformation for A_26_&A_11_. Interestingly, A_26_ and A_23_ adopt 2 different pathways to undergo transition from *+syn* to -*syn*. In the former, it happens through *cis* conformation (anti-clockwise rotation around the *glycosyl* bond), while in the latter, it happens via *trans* conformation (clockwise rotation). Both A_5_…A_32_&A_8_…A_29_ take-up +*syn…high-anti* (S12 Fig) via back-bone rearrangement (not via base flipping) and thus, retain the N6(A)…N7(A) hydrogen bond.

Intriguingly, 8 out of 10 G’s adopt -*syn* conformation (S13 Fig). In fact, in one of the strands, all the G’s (G_21_,G_24_,G_27_,G_30_&G_33_) adopt -*syn* conformation. This is associated with (ε,ζ,α,γ) favoring (*g* ^*-*^,*g* ^*+*^,*g* ^*+*^,*trans*) (>70%) at the GC step (S14 (I-L) Fig). While AG step tends to favor B-form geometry (>75%) (S14 (E-H) Fig), CA step has equal prevalence for both BI and BIII (S14 (A-D) Fig).

As before, this reflects in the helical twists with CA(9(16°)) and AG(11(9°)) steps confined to lower values (including negative values), while GC step taking a higher twist (31(10°)), causing frequent left-handedness in the helix (Fig. 6 (bottom), S15 Fig). These indicate the periodic occurrence of B-Z junction in d(CAG)_6_.d(CAG)_6_. Above conformational rearrangements result in a high RMSD of ~8Å at the end of the simulation (S16 Fig). Further, similar to above (S10 Fig), B-Z junction results in minor groove of the duplex occupied with more water and ion molecules compared to the major groove (S17 Fig), a characteristic of the Z-DNA.

Thus, it is clear that A…A mismatch favors *(±)syn…high-anti/(-)syn* conformation over *anti…anti* and +*syn…anti glycosyl* conformation and invokes B-Z junction. Formation of B-Z junction takes place either through base flipping or through backbone flipping without affecting the canonical G…C and C…G hydrogen bonding pattern (S18 Fig).

### Canonical (CTG)_6_.(CAG)_6_ duplex retains B-form

**Fig 7:**
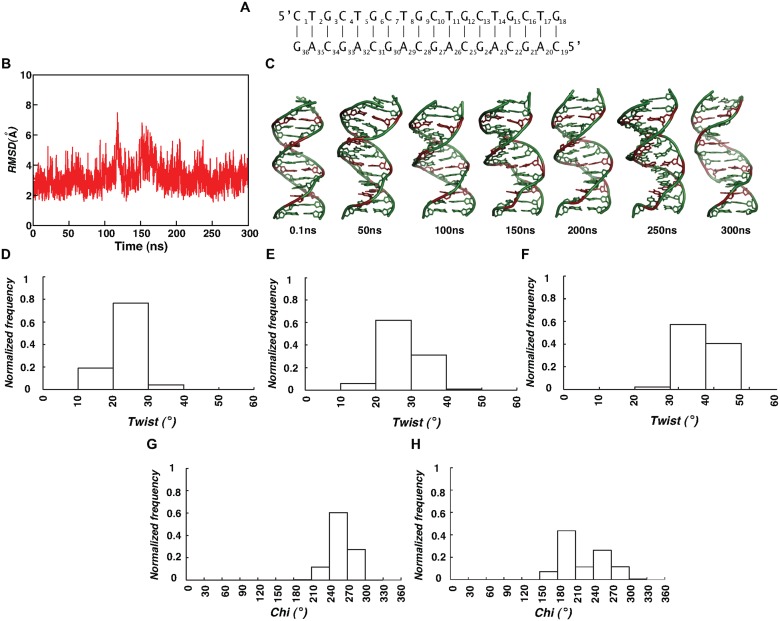
Canonical base pairs favor B-DNA structure for d(CTG)_6_.d(CAG)_6_ duplex. (A) 18mer DNA duplex used for the MD simulation. (B) Time vs RMSD profile showing confinement to starting B-form geometry. (C) Cartoon diagram showing compact B-DNA structures observed at various time intervals during the simulation. Note that A-T pairs are colored pink. Histograms illustrating twist angle preference during last 10ns at (D) 5’CT/5’AG, (E) 5’TG/5’CA and (F) 5’GC/5’GC steps. *Glycolsyl* conformation (calculated for last 10ns) of (G) A’s & (H) G’s showing the preference for *anti* conformation.

RMSD (~3.3 (0.9) Å) calculated over 300ns MD simulation of (CTG)_6_.(CAG)_6_ duplex (Fig. 7A) indicates that the molecule undergoes minimal conformational rearrangement from the starting B-form geometry (Fig. 7B). Strikingly, the overall structure doesn’t show any tendency to adopt Z-form, as can be visualized from Fig. 7C. Instead, it retains the compact B-form geometry.

The helical twist always stays positive (Fig. 7D-F), adopting a trend of high helical twists at GC (38.8(4°)) step compared to CT (23(4°)) and TG (28(5°)) steps over the last 10ns (S19 Fig). Unlike before, both A’s and G’s don’t favor *±syn* conformation and have the tendency to retain *anti glycosyl* conformation (180–270°) (~%70) (Fig. 7G,H). Significant conformational changes in the backbone are also not observed as (ε,ζ) fall profoundly in BI (*t*,*g* ^*-*^) or BII (*g* ^*-*^,*t*) conformation (S20 Fig). Similarly, (α&γ) favor either (*g-*,*g+*) or (*g-*,*t*). All these together pinpoint B-DNA conformational preference for (CTG)_6_.(CAG)_6_ duplex.

## Discussion

### A…A mismatch propels Z-DNA conformation

Structural information about the distortions caused by A…A mismatch in a DNA duplex is not yet well defined at the atomistic level. The only structure that has been reported so far with A…A mismatch in a DNA is the complex of a DNA duplex and Muts, an *E*. *coli* mismatch repair protein, with a significant bending at the mismatch site (PDB ID: 2WTU). NMR and thermodynamic studies of A…A mismatch containing DNA duplex offer controversial results. While some of them suggest that A…A mismatch destabilizes[18,19,20,21] the DNA duplex significantly, the others do not[22]. Physicochemical studies indicate that A…A mismatch in a GAC repeat adopt several distinct conformations in solution including Z-DNA[23,24]. In fact, it has been suggested that A…A mismatch in GAC repeat promotes Z-DNA formation [23].

Understanding the structural role of A…A mismatch is very important in the context of Huntington’s disorder and several spinocerebellar ataxias due to the formation of hairpin structures consisting of noncanonical A…A base-pairs. MD simulations carried out in this context reveal a very exquisite observation that A…A mismatch in a CAG repeat induces change in the helicity from right-handed B-DNA to left-handed Z-DNA. Even a single A…A mismatch tends to form a local Z-DNA structure leading to Z-DNA sandwich (Figs. 1,3). When the A…A mismatches occur in a regular interval, it leads to local left-handed Z-DNA formation at the mismatch site followed by a right-handed DNA at the canonical WC pair site leading to periodic B-Z junctions (Figs. 5,6). Formation of Z-DNA structure is evident from the preference for *(±)syn…high-anti/(-)syn glycosyl* conformation by A…A mismatch and backbone conformational angles (ε,ζ,α,γ) favoring (*g* ^*-*^,*g* ^*+*^,*g* ^*+*^,*t*), (*g* ^*-*^,*g* ^*-*^,*g* ^*+*^,*t*) and (*g* ^*-*^,*g* ^*-*^,*g* ^*-*^,*g* ^*+*^) at & around the mismatch site. Additionally, G’s prefer -*syn* conformation. This results in a low helical twist at the CA and AG steps in the midst of high twist at the GC step, a characteristic of B-Z junction (PDB ID 1FV7).

### Mechanism of formation of B-Z junction

An intriguing observation is that a single hydrogen bonded noncanonical A…A mismatch induces Z-DNA conformation through ‘zipper mechanism’ [25] assisted by base extrusion, base and/or backbone flipping (Figs. 1,6 and S2,S3&S21 Figs). While the sugar-phosphate backbone flipping is prominent in *anti…anti glycosyl* conformation, base extrusion and sugar-phosphate & base flipping are favored by *+syn…anti* conformation to transit from B-to-Z form DNA. Yet another interesting fact is that the above-mentioned Z-DNA formation is a noninstantaneous event, rather it propagates in a stepwise manner (Figs. 5I, 6 (Bottom) and S7 Fig). Though the noncanonical A…A mismatch impels Z-DNA conformation, the canonical base pairs have the prevalence for B-form geometry resulting in B-Z junction. Formation of such B-Z junction can be readily visualized by unwinding of the double helix irrespective of the starting *glycosyl* conformation (S22 Fig).

### Base flipping mechanism

A…A mismatch adopts 2 different ‘base flipping’ pathways to undergo transition from +*syn…anti* to -*syn…-syn* (Fig. 6) accompanied by sugar phosphate rearrangements. One mode of transition is *+syn* moving to -*syn* through *cis* conformation (via counter-clockwise rotation around *glycosidic* bond), while the other is via *trans* conformation (through clockwise rotation around the *glycosidic* bond). In general, DNA with *+syn…anti* conformation takes longer time to undergo the B-Z transition, compared to *anti…anti* conformation.

### Base pair nonisomorphism is the key factor for inducing Z-DNA conformation by A…A mismatch

Reported structural changes provoked by A…A mismatch can be attributed to the higher degree of nonisomorphism between A…A mismatch and the canonical base pairs. This can be visualized from the larger value of residual twist and radial difference [17,26], the measures of base pair nonisomorphism (S23 Fig). In fact, both residual twist (16º) and radial difference (1.6Å) are quite prominent for A…A mismatch with *anti…anti glycosyl* conformation, but, only residual twist (16º) is significant and the radial difference is negligible (0.2Å) in the case of +*syn…anti glycosyl* conformation. This may be the reason for the reluctance of A…A mismatch to retain *anti…anti* conformation and the transition to -*syn…-syn* being quite fast compared to *+syn…anti* starting conformation.

In general, the transition from B-to-Z involves complex mechanisms and exhibits a high-energy barrier to transit to Z-DNA conformation. In fact, several mechanisms have been proposed for B-to-Z transition[27] and a recent adaptively biased and steered MD study demonstrates the coexistence of zipper and stretch-collapse mechanisms engaged in transition[28]. However, the mechanistic effect that arises from the intrinsic extreme nonisosterecity of A…A mismatch with the canonical base pairs immediately dictates B-to-Z transition without the influence of any external factors. As the A…A mismatch is single hydrogen bonded, it exhibits enormous flexibility for base extrusion and flipping, facilitating the formation of Z-DNA through zipper mechanism. Interestingly, such a conformational change is not seen in the crystal structure of RNA duplex with A…A mismatch[13]. Thus, it is clear that the effect of A…A nonisomorphism is pronounced in the DNA and not in the RNA.

Several experimental studies have revealed that d(GA) [29], d(GAA) [30], d(GGA) [31] and d(GAC) [23,24] repeats that contain A…A mismatches are prone to adopt parallel homoduplex. Such preponderance for parallel duplex by these sequences may be due to left-handed Z-DNA provoking nature of A…A mismatch, which is a high-energy conformation. *Hitherto*, this aspect is not realized as there is no DNA duplex structure with A…A mismatch available with any sequence context. Earlier low-resolution 1D NMR studies on DNA duplexes comprising of A…A mismatch[18,19,20,21,22] offer only minimal information with some of them indicating notable destabilization induced at A…A mismatch site[18,19,20,21]. Strikingly, it has been shown by circular dichroism study that CAG repeat spectra resembles GA homoduplex but not CCG and CTG[32]. Propensity of A…A mismatch containing DNA to adopt a parallel DNA duplex is also reported[21]. However, the possibility of CAG repeat expansion to favor parallel duplex can be ruled out as it forms hairpin structure[7,8], which eventually leads to antiparallel orientation for the two strands of the DNA hairpin stem. Thus, DNA hairpin stems containing CAG repeat may adopt local Z-DNA conformation at A…A mismatch site leading to ‘B-Z junction’ as revealed by the current investigation. Our result gains support from earlier surface probing using anti-DNA antibody that demonstrated the presence of Z-DNA structure in CAG & CTG repeat expansions [33]. It can also be recalled that formation of hairpin structure with such Z-DNA stem has been observed earlier in a different sequence context [34,35,36]. Thus, we envisage that such noncanonical ‘B-Z junction’ in CAG repeat expansion may be one of the factors responsible for the newly emerging mechanism of ‘DNA toxicity’ observed in CAG repeat expansion[37].

Thus, for the first time it has been shown here that the A…A mismatch in a DNA duplex with CAG repeat is an inducer of local Z-form conformation through ‘zipper mechanism’ that stems from backbone flipping and base pair extrusion & flipping leading to B-Z junction. Such B-Z junction instilled by A…A mismatch results from the mechanistic effect intrinsic to the nonisoterecity of A…A mismatch with the flanking canonical base pairs. With emergence of evidence on ‘DNA toxicity’ of CAG overexpansion and its role in triggering cell death [9,10], one can envision that occurrence of B-Z junction is the molecular basis for Huntington’s disorder and several spinocerebellar ataxias. This further leads to the speculation that B-Z junction binding protein may have a role in the diseased states. Reported results would further be useful in understanding DNA repair mechanisms involving A…A mismatch, thus adding a new dimension to the role of A…A nonisosterecity on DNA structure.

## Methods

### Modeling of DNA duplex with A…A mismatch

Initially, (CTG.CAG)_5_ & (CTG.CAG)_6_ DNA duplexes containing canonical C…G and G…C base-pairs with ideal B-form geometry are generated using 3DNA[38]. These models are subsequently manipulated to introduce a non-canonical A…A mismatch in the middle of canonical base pairs to generate a 15mer DNA duplex (Fig. 1A) using Pymol (www.pymol.org, Schrödinger, LLC) molecular modeling software. A…A mismatch is modeled so as to form N6(A)…N1(A) hydrogen bond. For the generation of model with periodic A…A mismatches (18mer, Fig. 3A), ‘T’s in the (CTG.CAG)_6_ duplex are replaced manually with A’s as mentioned above. To establish base-sugar connectivity and to restraint the sugar-phosphate backbone conformation, the models are refined using X-PLOR [39] by constrained-restrained molecular geometry optimization and van der Waals energy minimization. The second conformation for the A…A mismatch, viz., N6(A)…N1(A) hydrogen bond with *+syn…anti glycosyl* conformation is generated using X-PLOR by applying appropriate restraints. Subsequently, the models are subjected to a total of 1.5μs molecular dynamics simulations (MD) using Sander module of AMBER 12 package [40].

### Molecular dynamics simulation protocol

X-PLOR generated duplex models with A…A mismatches and the 3DNA generated canonical (CTG.CAG)_6_ duplex are solvated with TIP3P water molecules and net-neutralized with Na^+^ counter ions. Following the protocols described in our earlier papers [17,41,42], equilibration and production runs are pursued for 300ns for the sequences given in Table 1. Simulations are performed under isobaric and isothermal conditions with SHAKE (tolerance = 0.0005 Å) on the hydrogens [43], a 2fs integration time and a cut-off distance of 9 Å for Lennard-Jones interaction. FF99SB forcefield is used and the simulation is carried out at neutral pH. Trajectories are analyzed using Ptraj module of AMBER 12.0. Helical parameters and conformation angles are extracted from the output of 3DNA using in-house programs. Due to the presence of noncanonical base pairs, helical twist angles are calculated with respect to C1’…C1’ vector [17,41,42]. Pymol is used for visualization and MATLAB software (The MathWorks Inc., Natick, Massachusetts, United States) is used for plotting the graphs.

**Table 1:** Sequences used for the 300ns MD simulation.

Sequence ID	Sequence	*Chi* angle for A…A mismatch
**1**	**5’CTGCTGCAGCTGCTG**	***anti…anti***
	| | | | | | | * | | | | | | |	
	**GACGACGACGACGAC 5’**	
**2**	**5’CTGCTGCAGCTGCTG**	***syn…anti***
	| | | | | | | * | | | | | | |	
	**GACGACGACGACGAC 5’**	
**3**	**5’CAGCAGCAGCAGCAGCAG**	***anti…anti***
	| * | | * | | * | | * | | * | | * |	
	**GACGACGACGACGACGAC 5’**	
**4**	**5’CAGCAGCAGCAGCAGCAG**	***syn…anti***
	| * | | * | | * | | * | | * | | * |	
	**GACGACGACGACGACGAC 5’**	
**5**	**5’CAGCAGCAGCAGCAGCAG**	***Not applicable***
	**| | | | | | | | | | | | | | | | | |**	
	**GTCGTCGTCGTCGTCGTC 5’**	

## Supporting Information

S1 Movie Formation of B-Z junction provoked by A_8_…A_23_ mismatch (colored pink) through backbone flipping in d(CAG)_2_CAG(CAG)_2_.d(CTG)_2_CAG(CTG)_2_ DNA duplex (Fig. 1A). Central heptamer of the duplex is shown. Note that A_8_…A_23_ mismatch is in *anti*…*anti* starting *glycosyl* conformation.
(MOV)

S2 Movie A_8_…A_23_ mismatch (with *anti*…*anti* starting *glycosyl* conformation) induced backbone flipping at the mismatch site leading to the formation of B-Z junction. (MOV)

S3 Movie Formation of B-Z junction provoked by A_8_…A_23_ mismatch (with *anti*…*anti* starting *glycosyl* conformation) in d(CAG)_2_CAG(CAG)_2_.d(CTG)_2_CAG (CTG)_2_ DNA duplex through backbone flipping. Note that the central 11mer is shown.
(MOV)

S4 Movie Formation of B-Z junction provoked by A_8_…A_23_ mismatch (colored pink) through backbone flipping in d(CAG)_2_CAG(CAG)_2_.d(CTG)_2_CAG(CTG)_2_ DNA duplex (Fig. 1A). Central heptamer of the duplex is shown. Note that A_8_…A_23_ mismatch is in *+syn*…*anti* starting *glycosyl* conformation.
(MOV)

S5 Movie Base flipping leading to the formation of B-Z junction at A_14_…A_23_ mismatch site in d(CAG)_6_.d(CAG)_6_ DNA duplex with *+syn…anti* starting conformation for the mismatch (Fig. 5A). Note that one of the A’s moves towards minor groove and undergoes flipping by rotating in counter-clockwise direction.
(MOV)

S6 Movie Base flipping leading to the formation of B-Z junction at A_11_…A_26_ mismatch site in d(CAG)_6_.d(CAG)_6_ DNA duplex with *+syn…anti* starting conformation for the mismatch (Fig. 5A). Note that prior to flipping, both the A’s are moving apart that results in total loss of hydrogen bond and subsequently, one of the A’s flips by rotating in clockwise direction.
(MOV)

S1 Fig Local unwinding of d(CAG)_2_CAG(CAG)_2_.d(CTG)_2_CAG(CTG)_2_ DNA duplex by A_8_…A_23_ mismatch and formation of Z-DNA sandwich. Comparison of B-Z junction formed by A_8_…A_23_ mismatch (**Top-Left & Top-middle, current study**) and by L-deoxy guanine and L-deoxy cytosine (**Top-Right, PDB ID: 1FV7, Lowest energy structure**). **(Bottom)** Sequence vs helical twist angle of the central 11-mer (Fig. 1A) corresponding to the average structure calculated over 99.9-100ns **(Bottom-Left)** and 299.9-300ns **(Bottom-middle)**. Note the low helical twist at the mismatch site. Similar trend is also seen in B-Z junction induced by L-deoxy guanine and L-deoxy cytosine (**Bottom-Right, PDB ID: 1FV7, Lowest energy structure**) leading to Z-DNA sandwich structure (calculated with respect to C1’…C1’ vector).
(TIF)

S2 Fig Hydrogen bond conformational dynamics at A_8_…A_23_ mismatch (Fig. 1A) with *anti…anti* starting *glycosyl* conformation. (**Top and Middle**) Snapshots showing the occurrence of different hydrogen bonding patterns during the simulation. Possibilities for N1(A_8_)…N6(A_23_) & N6(A_23_)…N3(A_8_) hydrogen bonds or total loss of hydrogen bond between A_8_ and A_23_ can also be seen. (**Bottom**) Time vs hydrogen bond distance profile for: (**Left**) N1(A_8_)…N6(A_23_) (black) & N6(A_8_)…N1(A_23_) (red), (**Middle**) O2(C_7_)…N2(G_24_) (black), N3(C_7_)…N1(G_24_) (red) & N4(C_7_)…O6(G_24_) (blue) and (**Right**) N2(G_9_)…O2(C_22_) (black), N3(G_9_)…N1(C_22_) (red) and O6(G_9_)…N2(C_22_) (blue) that correspond to A_8_…A_23_, C_7_…G_24_ and G_9_…C_22_ base pairs respectively. Note the equal preference for N1(A_8_)…N6(A_23_) (black) & N6(A_8_)…N1(A_23_) (red) hydrogen bonds after 150ns for A_23_…A_8_.
(TIF)

S3 Fig *Glycosyl* conformation and hydrogen bonding associated with local Z-DNA formation for d(CAG)_2_CAG(CAG)_2_.d(CTG)_2_CAG(CTG)_2_ duplex with A_8_…A_23_ in *+syn…anti* starting *glycosyl* conformation. (**A**) Time vs *chi* angle profile for (**Left**) G_9_&A_11_ and (**Right**) G_21_,A_23_&G_24_ bases. Note the preference for -*syn glycosyl* conformation for G_9_,A_11_,G_21_&G_24_ and *+syn glycosyl* conformation for A_23_ (**B**) Time vs hydrogen bond distance profile for: (**Left**) N6(A_23_)…N1(A_8_) (black) & N7(A_23_)…N6(A_8_) (red), (**Middle**) O2(C_7_)…N2(G_24_) (black), N3(C_7_)…N1(G_24_) (red) & N4(C_7_)…O6(G_24_) (blue) and (**Right**) N2(G_9_)…O2(C_22_) (black), N3(G_9_)…N1(C_22_) (red) and O6(G_9_)…N2(C_22_) (blue) that correspond to A_8_…A_23_, C_7_…G_24_ and G_9_…C_22_ base pairs respectively. Note the total loss of hydrogen bonds after 150ns for A_23_…A_8_ (**Left**) that arises due to the stacked conformation of A_23_&A_8_, while the canonical C_7_…G_24_ and G_9_…C_22_ retain their hydrogen bonds. (**C-E**) Different hydrogen bonding patterns observed for A_8_…A_23_ during the simulation. Note the total loss of hydrogen bond in (**E**) that happens between 30-40ns.
(TIF)

S4 Fig Influence of A_8_…A_23_ mismatch on the sugar-phosphate backbone conformation. 3D plots showing the relationship between ε & ζ and α & γ with respect to time in the case of d(CAG)_2_CAG(CAG)_2_.d(CTG)_2_CAG(CTG)_2_ duplex with *+syn…anti glycosyl* starting conformation. The corresponding step is indicated on top of the 3D plot.
(TIF)

S5 Fig Formation of Z-DNA sandwich structure and unwinding of the double helix with A_8_…A_23_ mismatch in +*syn…anti* starting *glycosyl* conformation. Sequence vs helical twist angle (central 11-mer) and the corresponding average structure (cartoon representation) calculated over (**A**) 0.09–0.1ns (**B**) 99.9-100ns (**C**) 149.9-150ns and (**D**) 299.9-300ns. Note that the low helical twists at and around the mismatch site are sandwiched between high helical twists (sequence vs twist profiles given in **A-D**). A_8_…A_23_ mismatch is colored pink and O4’ atoms of the sugars are colored orange in the cartoon representation of the average structures. Dotted lines indicate the helical twist angle corresponding to ideal B-form. Note the unwinding of the double helix around the mismatch site.
(TIF)

S6 Fig 2D plots indicating backbone conformational preference for d(CAG)_2_CAG(CAG)_2_.d(CTG)_2_CAG(CTG)_2_ duplex with A_8_…A_23_ in *+syn…anti* starting *glycosyl* conformation during the last 10ns. (ε&ζ) and (α&γ) 2D plots corresponding to the first strand is given in 2^nd^ and 3^rd^ columns respectively along with the appropriate step marked in the 1^st^ column. (ε&ζ) and (α&γ) 2D plots corresponding to the second strand is given in 5^th^ and 6^th^ columns respectively along with the appropriate step marked in the 4^th^ column.
(TIF)

S7 Fig Transition from B- to Z-DNA. Snapshots showing transition from B- to Z-DNA through sugar-phosphate conformational rearrangement at and around A_8_…A_23_ mismatch (colored pink) in d(CAG)_2_CAG(CAG)_2_.d(CTG)_2_CAG(CTG)_2_ duplex with *+syn…anti* starting *glycosyl* conformation.
(TIF)

S8 Fig Helical twist angles reflecting the characteristic of B-Z junction in a d(CAG)_6_.d(CAG)_6_ duplex with A…A mismatch in *anti…anti* starting *glycosyl* conformation. Sequence vs helical twist angle calculated for the average structure over last 100ps showing a high twist at GC step and a low twist at CA and AG steps.
(TIF)

S9 Fig Contour density plot indicating backbone conformational preference for d(CAG)_6_.d(CAG)_6_ duplex with *anti…anti* starting *glycosyl* conformation for A…A mismatch. (ε&ζ) and (α&γ) contour density plot corresponding to (**A-D**) CA, (**E-H**) AG & (**I-L**) GC steps. Note that the first two columns belong to residues from C_1_ to G_18_ of the duplex, while the third and fourth belong to the complementary residues (C_19_ to G_36_) of the duplex. While the first and third columns indicate the relationship between ε & ζ (ε in X-axis and ζ in Y-axis), the second and fourth columns illustrate the relationship between α & γ (α in X-axis and γ in Y-axis). Scaling used for contour density plot is shown in the 4^th^ row. Note the strong preponderance for Z-form geometry by CA and GC steps.
(TIF)

S10 Fig Ion (top) and water (bottom) density around d(CAG)_6_.d(CAG)_6_ duplex with *anti…anti* starting *glycosyl* conformation for A…A mismatch. Note that the minor groove (100ns, 200ns, 300ns) is highly solvated compared to the major groove.
(TIF)

S11 Fig Snapshots showing in detail about base flipping at A_23_…A_14_ mismatch site. The corresponding simulation time scale is mentioned below the mismatch.
(TIF)

S12 Fig *+Syn…high-anti glycosyl* conformational preference for A…A mismatches in d(CAG)_6_.d(CAG)_6_ duplex with *+syn…anti* starting *glycosyl* conformation. Time vs *chi* profile for A_32_…A_5_ (**Top**) and A_29_…A_8_ (**Bottom**) mismatches showing the transition from *+syn…anti* to *+syn…high-anti* conformation.
(TIF)

S13 Fig Time vs *chi* profile for G’s in d(CAG)_6_.d(CAG)_6_ duplex with *+syn…anti glycosyl* starting conformation for A…A mismatches. Time vs *chi* angle profile for G’s indicating the preponderance for -*syn* conformation (except G_6_ and G_9_) favoring Z-DNA conformation.
(TIF)

S14 Fig Contour density plot indicating backbone conformational preference for d(CAG)_6_.d(CAG)_6_ duplex with *+syn…anti* starting *glycosyl* conformation for A…A mismatches. (ε&ζ) and (α&γ) contour density plot corresponding to CA (**A-D**), AG (**E-H**) & GC (**I-L**) steps. Note that the first two columns belong to one of the strands of the duplex (C_1_ to G_18_), while the third and fourth columns belong to the complementary second strand of the duplex (C_19_ to G_36_). While the first and third columns indicate the relationship between ε & ζ (ε in X-axis and ζ in Y-axis), the second and fourth columns illustrate the relationship between α & γ (α in X-axis and γ in Y-axis). Scaling used for contour density plot is shown in the 4^th^ row. Note the strong preponderance for Z-form geometry by GC step (viz., more than 70% of (ε,ξ,α,γ) in (*g* ^*-*^,*g* ^*+*^,*g* ^*+*^,*t*) conformation). CA step as well shows the tendency for Z-form geometry with (ε,ξ,α,γ) in (*g* ^*-*^,*g* ^*-*^,*g* ^*-*^,*g* ^*+*^) conformation. AG step favors B-form geometry with ~59% of (*t*,*g* ^*-*^,*g* ^*+*^,*t*), ~23% of (*t*,*g* ^*-*^,*g* ^*-*^,*t*) and 18% of (*t*,*g* ^*-*^,*g* ^*-*^,*g* ^*+*^) for (ε,ξ,α,γ).
(TIF)

S15 Fig Helical twists corresponding to d(CAG)_6_.d(CAG)_6_ duplex with *+syn…anti* starting *glycosyl* conformation for A…A mismatches. (**Top**) Histogram of twist angles calculated over 291-300ns. (**Bottom**) Sequence vs. twist angle corresponding to the average structure calculated over last 100ps. Note the low twist at the CA & AG steps and high twist at the GC step. Though CA step takes wide range of helical twist (between -20º to +50º), it has preference for low twist in the range of -20 to +20 (~70%).
(TIF)

S16 Fig Time vs RMSD profile corresponding to d(CAG)_6_.d(CAG)_6_ duplex with *+syn…anti* (black) and *anti…anti* (red) starting *glycosyl* conformation for A…A mismatch. Note that while the latter attains the RMSD of ~8 Å very early in the simulation, the former attains the RMSD of ~8 Å only ~200ns as indicated by solid arrows. Dotted double-headed arrows indicate two ensembles of structures in the case of *+syn…anti* starting *glycosyl* conformation: one with RMSD of ~5 Å during 200ns and other with RMSD of ~8 Å beyond 200ns.
(TIF)

S17 Fig Ion (top) and water (bottom) density around d(CAG)_6_.d(CAG)_6_ duplex with *+syn…anti* starting *glycosyl* conformation for A…A mismatch. Note that the minor groove (100ns, 200ns, 300ns) is highly solvated compared to the major groove.
(TIF)

S18 Fig Histogram corresponding to the canonical G…C and C…G hydrogen bonding distance of d(CAG)_6_.d(CAG)_6_ duplex with (A&B) *anti…anti* and (C&D) *+syn…anti* starting *glycosyl* conformation. Note that the normalized frequency (Y-axis) is represented against hydrogen bonding distance (X-axis) over the last 10ns of the 300ns simulation.
(TIF)

S19 Fig Sequence vs helical twist angles calculated for the average structure (last 100ps) corresponding to d(CTG)_6_.d(CAG)_6_ duplex with canonical base pairs. Note that unlike in S8&S15 (Bottom) Figs, the helical twist stays close to 30° indicative of geometry close to B-DNA.
(TIF)

S20 Fig B-DNA like backbone conformational preference for d(CTG)_6_.d(CAG)_6_ duplex. (ε&ζ) and (α&γ) 2D contour density plots corresponding to **(Top)** 5’CT/5’AG, **(Middle)** 5’TG/5’CA and **(Bottom)** 5’GC/5’GC steps. Note that (ε&ζ) does not exhibit any other conformational preference apart from BI (83%) and BII (17%). Similarly, as in the B-form, (α&γ) favor (*g-*, *g+*) or (*g+*, *t*) conformations. Exceptionally, at the TG step, (α&γ) also favor (*g-*, *t*) conformation, which is also favored by B-DNA. First two columns belong to one of the strands of the duplex (C_1_ to G_18_), while the third and fourth columns belong to the complementary second strand of the duplex (C_19_ to G_36_). While the first and third columns indicate the relationship between ε & ζ (ε in X-axis and ζ in Y-axis), the second and fourth columns illustrate the relationship between α & γ (α in X-axis and γ in Y-axis). Scaling used for contour density plot is shown in the 4^th^ row.
(TIF)

S21 Fig Base extrusion observed at the A…A mismatch site (colored red) leading to the formation of Z-DNA observed in d(CAG)_6_.d(CAG)_6_ duplex with A…A mismatch in *anti…anti* starting *glycosyl* conformation. Note that only the pentamer sequence is shown for clarity.
(TIF)

S22 Fig Average structures at 300ns (calculated over last 100ps) illustrating the characteristic of B-Z junction enforced by A…A mismatch. Cartoon representation of central hexamer corresponding to d(C_7_X_8_G_9_C_10_X_11_G_12_).d(C_25_A_26_G_27_C_28_A_29_G_30_), wherein X = T for canonical duplex **(Top)** and X = A for non-canonical duplex (**Middle and Bottom**). A…A mismatch with *anti…anti* and *syn…anti glycosyl* starting conformations are shown in the **middle** and **bottom** respectively. Note the smooth right-handedness in canonical duplex, whereas, the A…A mismatch induced B-Z junction leads to opening of the double helix.
(TIF)

S23 Fig Superposition of canonical G…C pair with noncanonical A…A mismatch showing the extent of base pair nonisomorphism. Residual twist and radial difference, the quantitative measures of base triplet nonisomorphism, are quite high between G…C and A…A (~16 is ~1.6Å), when the latter is in *anti…anti glycosyl* conformation **(Top)**. When A…A is in *syn…anti* *glycosyl* conformation only the residual twist is quite high and the radial difference is negligible (~16 is ~0.2Å).
(TIF)
