# Supplementary figures and images for: Twisting Right to Left: A…A Mismatch in a CAG Trinucleotide Repeat Overexpansion Provokes Left-Handed Z-DNA Conformation
Source: PLoS Comput Biol. 2015 Apr 13;11(4):e1004162. doi: 10.1371/journal.pcbi.1004162 (PMC4395422; doi:10.1371/journal.pcbi.1004162)

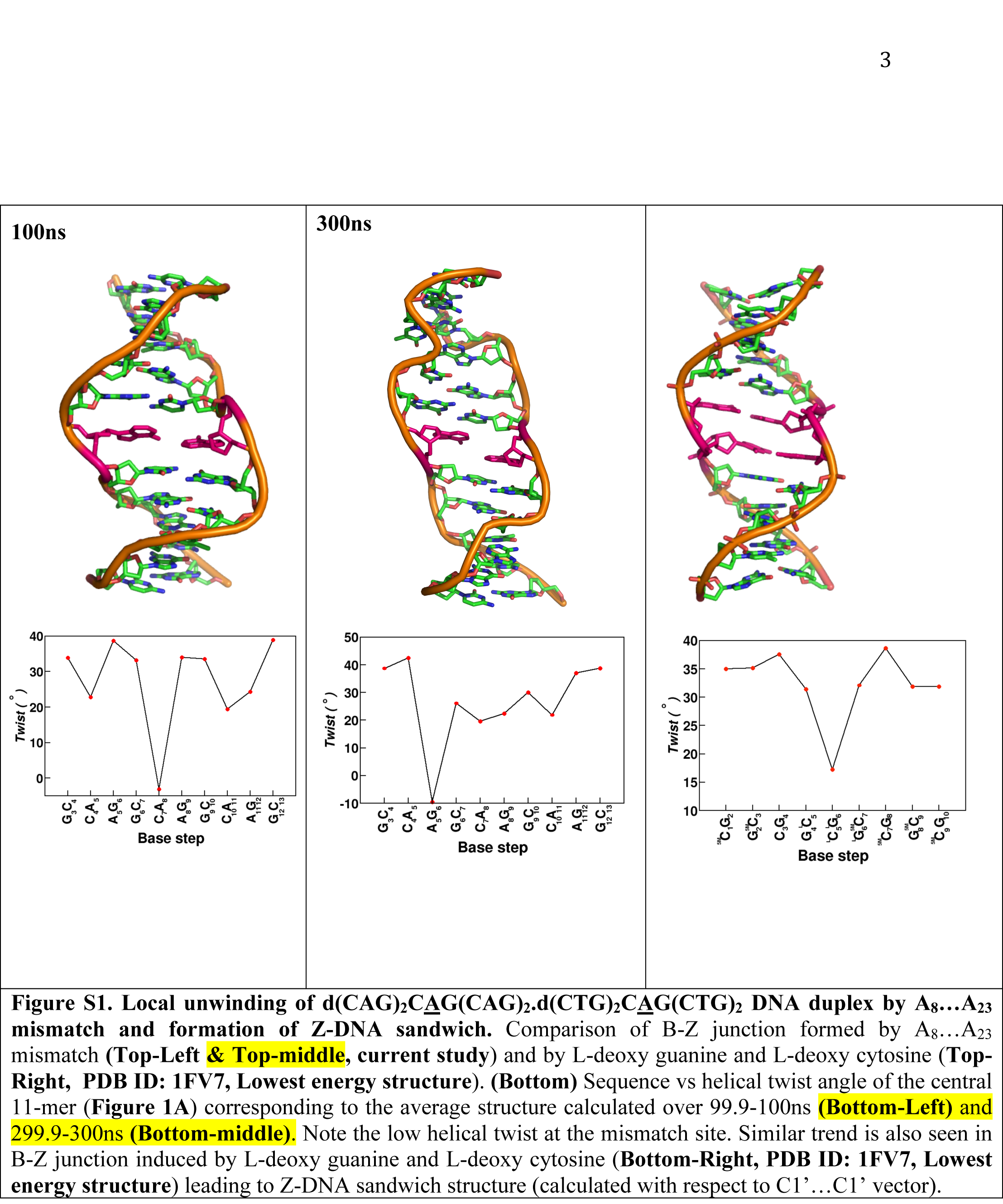

Supplement: S1 Fig — Comparison of B-Z junction formed by A8…A23 mismatch (Top-Left & Top-middle, current study) and by L-deoxy guanine and L-deoxy cytosine (Top-Right, PDB ID: 1FV7, Lowest energy structure). (Bottom) Sequence vs helical twist angle of the central 11-mer (Fig. 1A) corresponding to the average structure calculated over 99.9-100ns (Bottom-Left) and 299.9-300ns (Bottom-middle). Note the low helical twist at the mismatch site. Similar trend is also seen in B-Z junction induced by L-deoxy guanine and L-deoxy cytosine (Bottom-Right, PDB ID: 1FV7, Lowest energy structure) leading to Z-DNA sandwich structure (calculated with respect to C1’…C1’ vector). (TIF) [file pcbi.1004162.s007.tif]

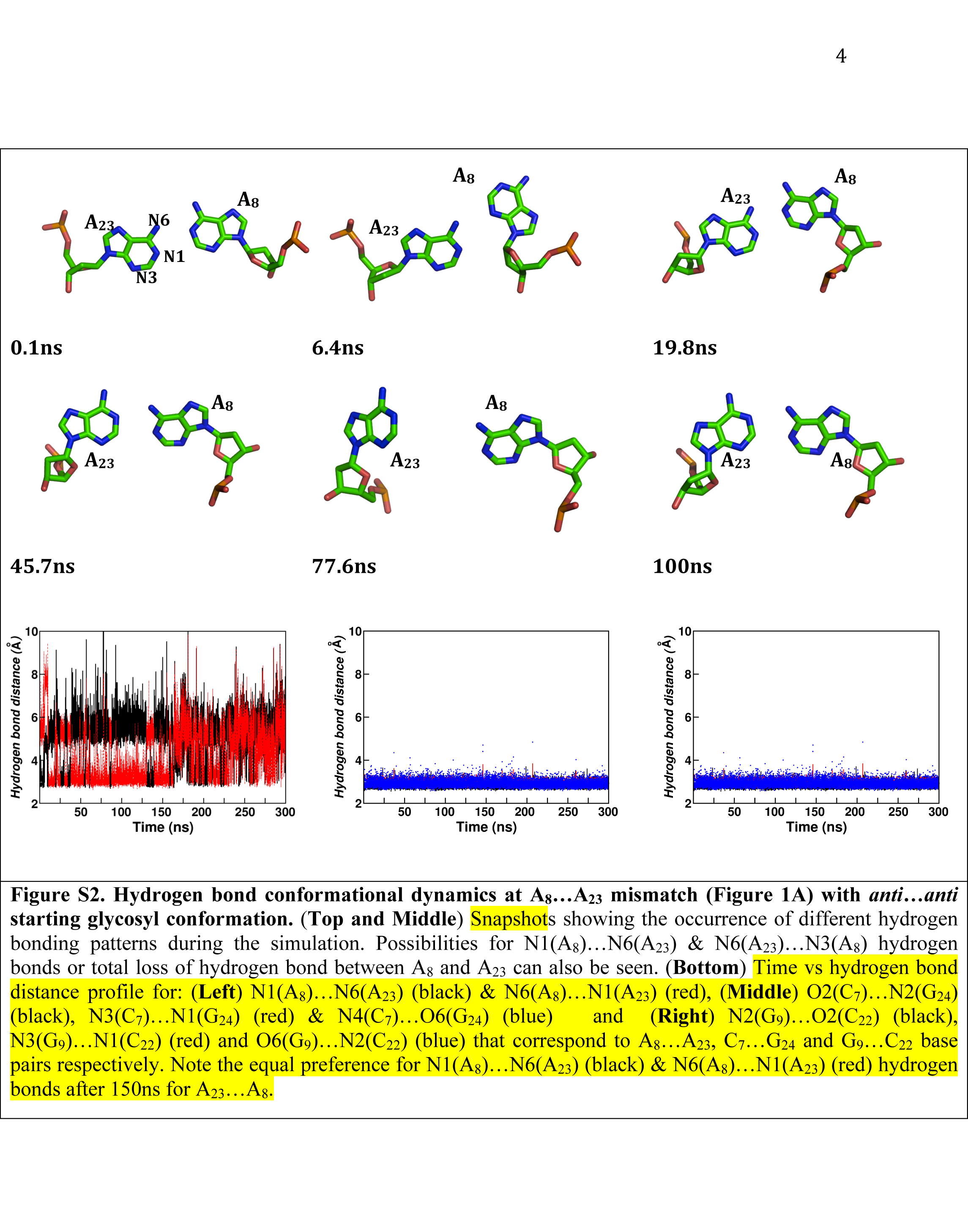

Supplement: S2 Fig — (Top and Middle) Snapshots showing the occurrence of different hydrogen bonding patterns during the simulation. Possibilities for N1(A8)…N6(A23) & N6(A23)…N3(A8) hydrogen bonds or total loss of hydrogen bond between A8 and A23 can also be seen. (Bottom) Time vs hydrogen bond distance profile for: (Left) N1(A8)…N6(A23) (black) & N6(A8)…N1(A23) (red), (Middle) O2(C7)…N2(G24) (black), N3(C7)…N1(G24) (red) & N4(C7)…O6(G24) (blue) and (Right) N2(G9)…O2(C22) (black), N3(G9)…N1(C22) (red) and O6(G9)…N2(C22) (blue) that correspond to A8…A23, C7…G24 and G9…C22 base pairs respectively. Note the equal preference for N1(A8)…N6(A23) (black) & N6(A8)…N1(A23) (red) hydrogen bonds after 150ns for A23…A8. (TIF) [file pcbi.1004162.s008.tif]

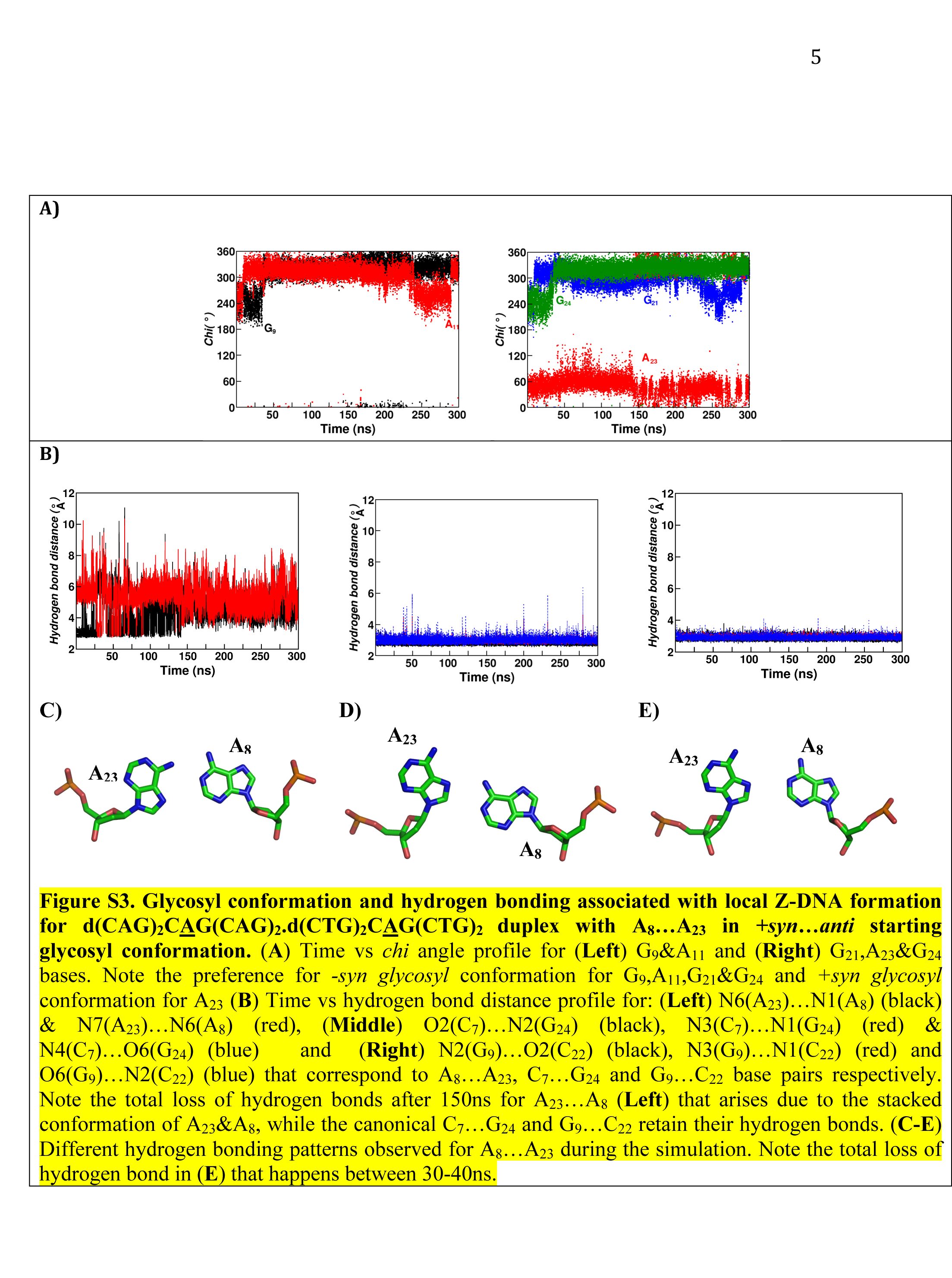

Supplement: S3 Fig — (A) Time vs chi angle profile for (Left) G9&A11 and (Right) G21,A23&G24 bases. Note the preference for -syn glycosyl conformation for G9,A11,G21&G24 and +syn glycosyl conformation for A23 (B) Time vs hydrogen bond distance profile for: (Left) N6(A23)…N1(A8) (black) & N7(A23)…N6(A8) (red), (Middle) O2(C7)…N2(G24) (black), N3(C7)…N1(G24) (red) & N4(C7)…O6(G24) (blue) and (Right) N2(G9)…O2(C22) (black), N3(G9)…N1(C22) (red) and O6(G9)…N2(C22) (blue) that correspond to A8…A23, C7…G24 and G9…C22 base pairs respectively. Note the total loss of hydrogen bonds after 150ns for A23…A8 (Left) that arises due to the stacked conformation of A23&A8, while the canonical C7…G24 and G9…C22 retain their hydrogen bonds. (C-E) Different hydrogen bonding patterns observed for A8…A23 during the simulation. Note the total loss of hydrogen bond in (E) that happens between 30-40ns. (TIF) [file pcbi.1004162.s009.tif]

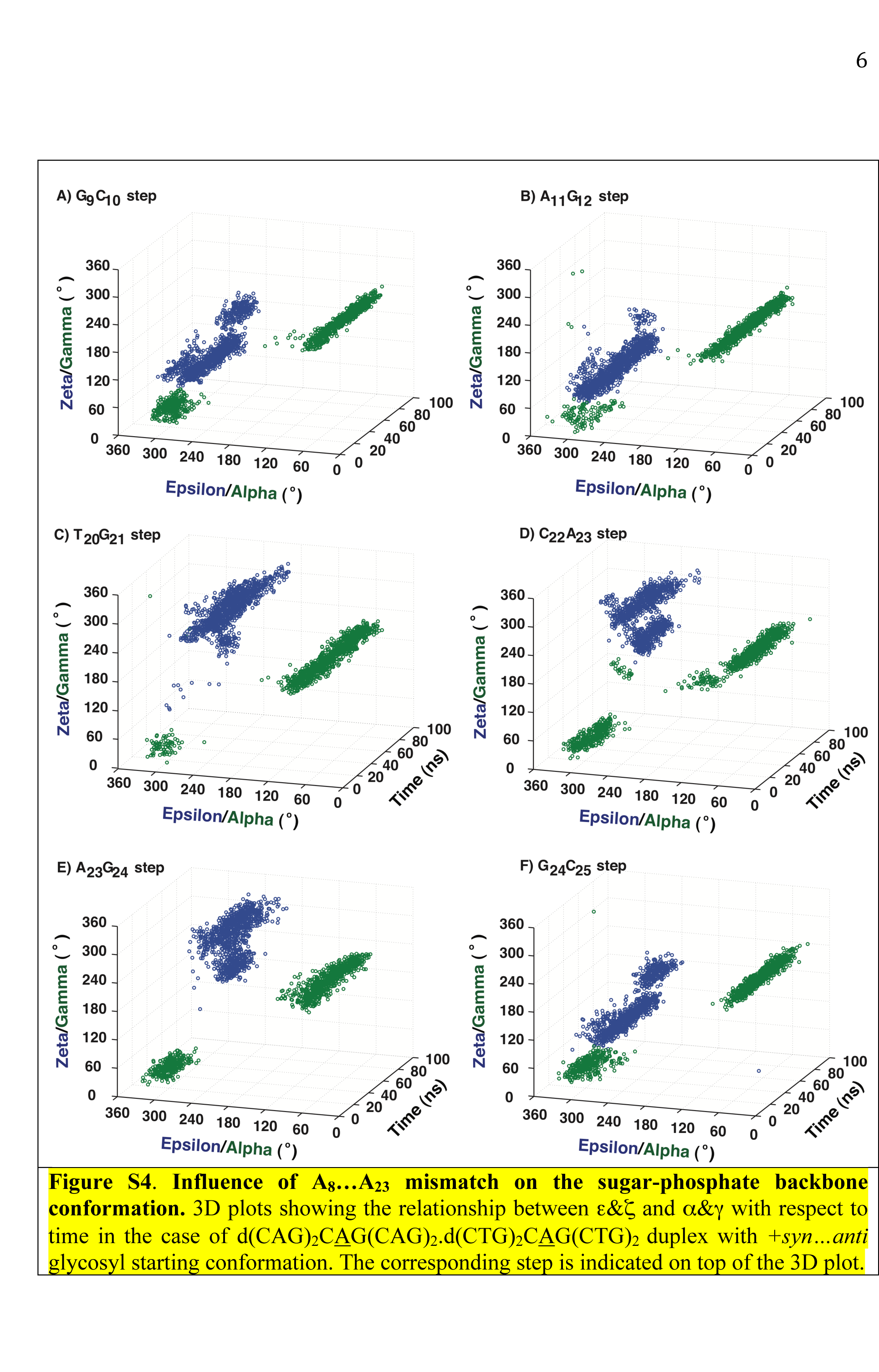

Supplement: S4 Fig — 3D plots showing the relationship between ε & ζ and α & γ with respect to time in the case of d(CAG)2CAG(CAG)2.d(CTG)2CAG(CTG)2 duplex with +syn…anti glycosyl starting conformation. The corresponding step is indicated on top of the 3D plot. (TIF) [file pcbi.1004162.s010.tif]

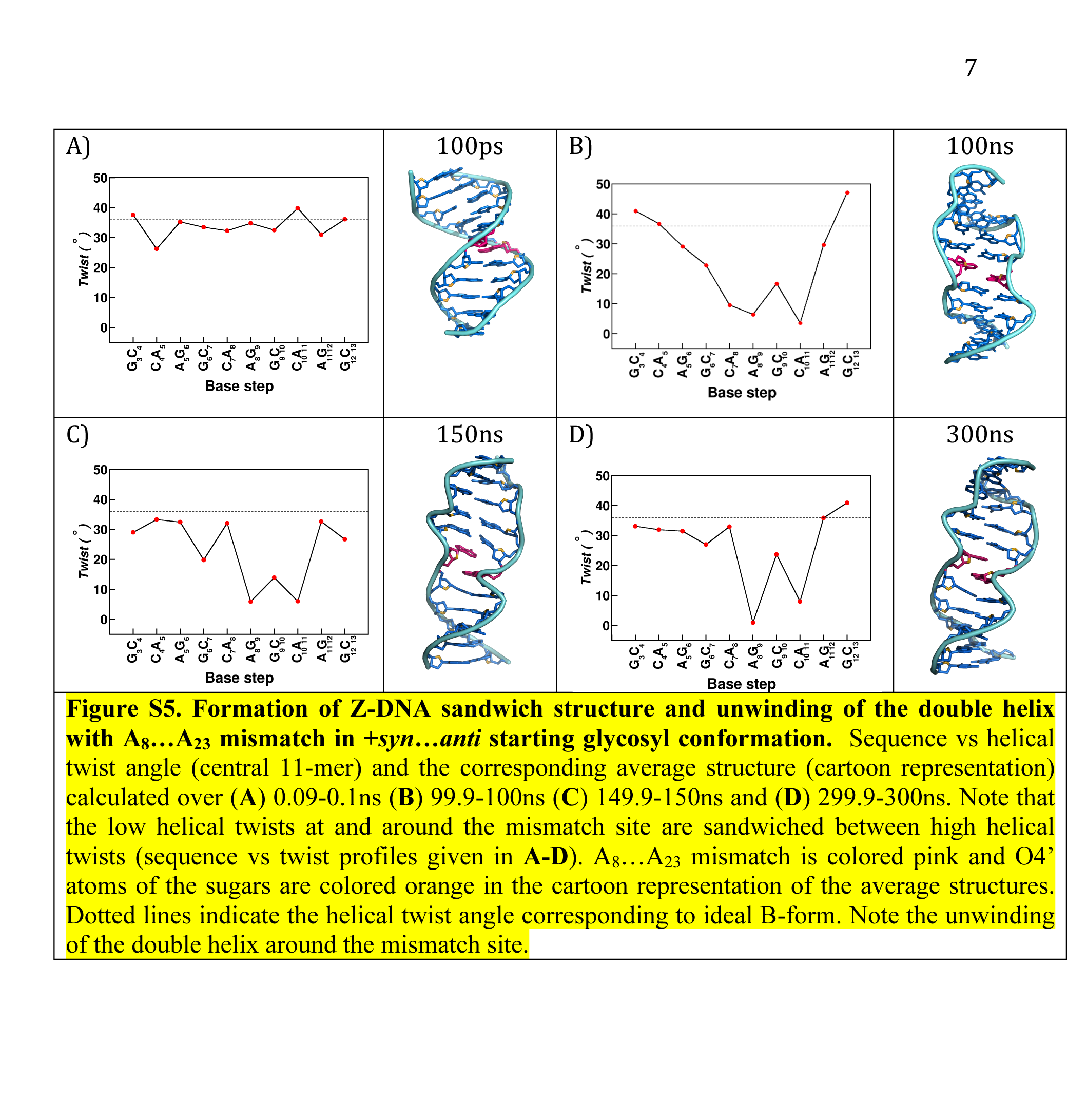

Supplement: S5 Fig — Sequence vs helical twist angle (central 11-mer) and the corresponding average structure (cartoon representation) calculated over (A) 0.09–0.1ns (B) 99.9-100ns (C) 149.9-150ns and (D) 299.9-300ns. Note that the low helical twists at and around the mismatch site are sandwiched between high helical twists (sequence vs twist profiles given in A-D). A8…A23 mismatch is colored pink and O4’ atoms of the sugars are colored orange in the cartoon representation of the average structures. Dotted lines indicate the helical twist angle corresponding to ideal B-form. Note the unwinding of the double helix around the mismatch site. (TIF) [file pcbi.1004162.s011.tif]

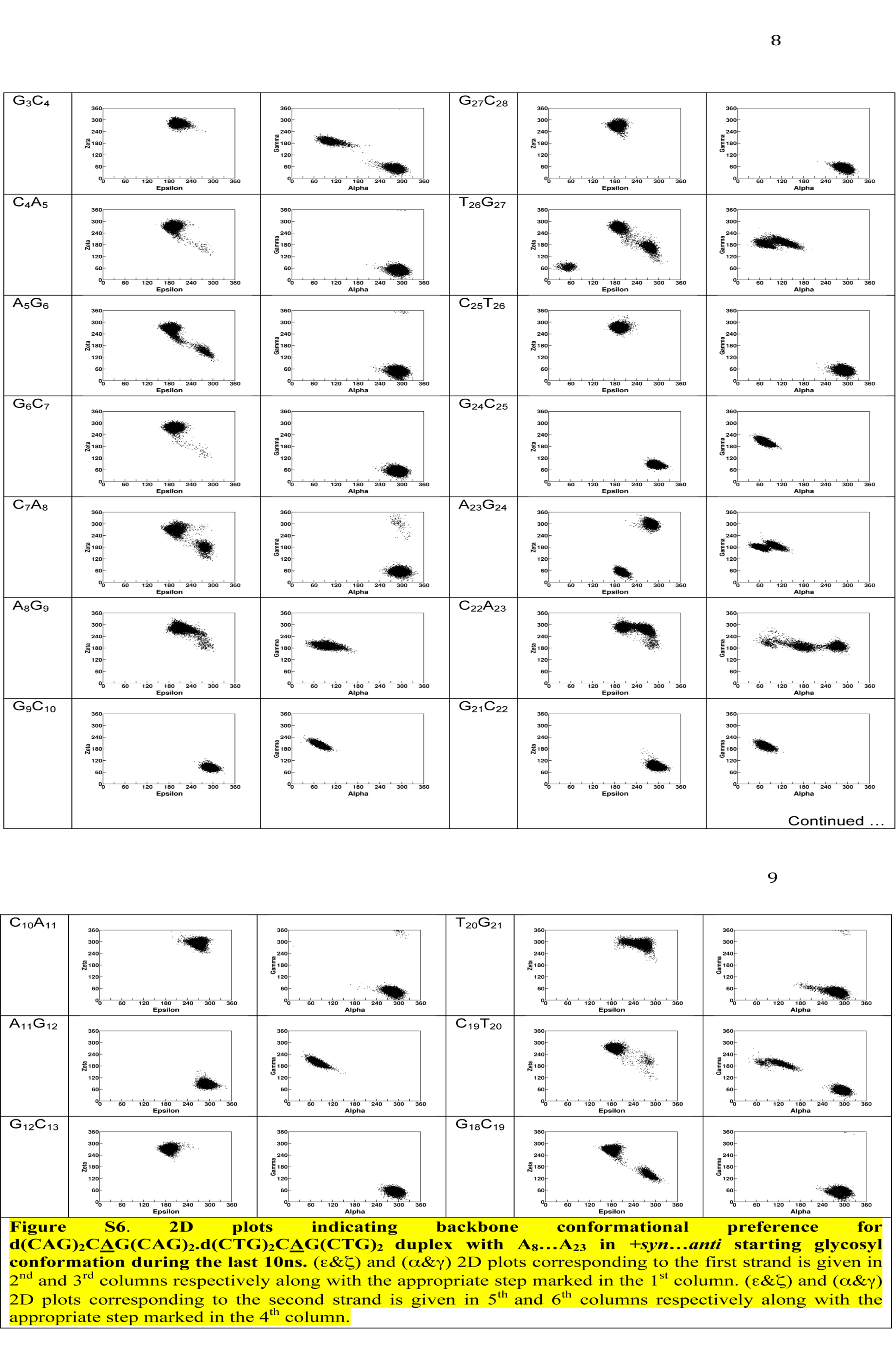

Supplement: S6 Fig — (ε&ζ) and (α&γ) 2D plots corresponding to the first strand is given in 2nd and 3rd columns respectively along with the appropriate step marked in the 1st column. (ε&ζ) and (α&γ) 2D plots corresponding to the second strand is given in 5th and 6th columns respectively along with the appropriate step marked in the 4th column. (TIF) [file pcbi.1004162.s012.tif]

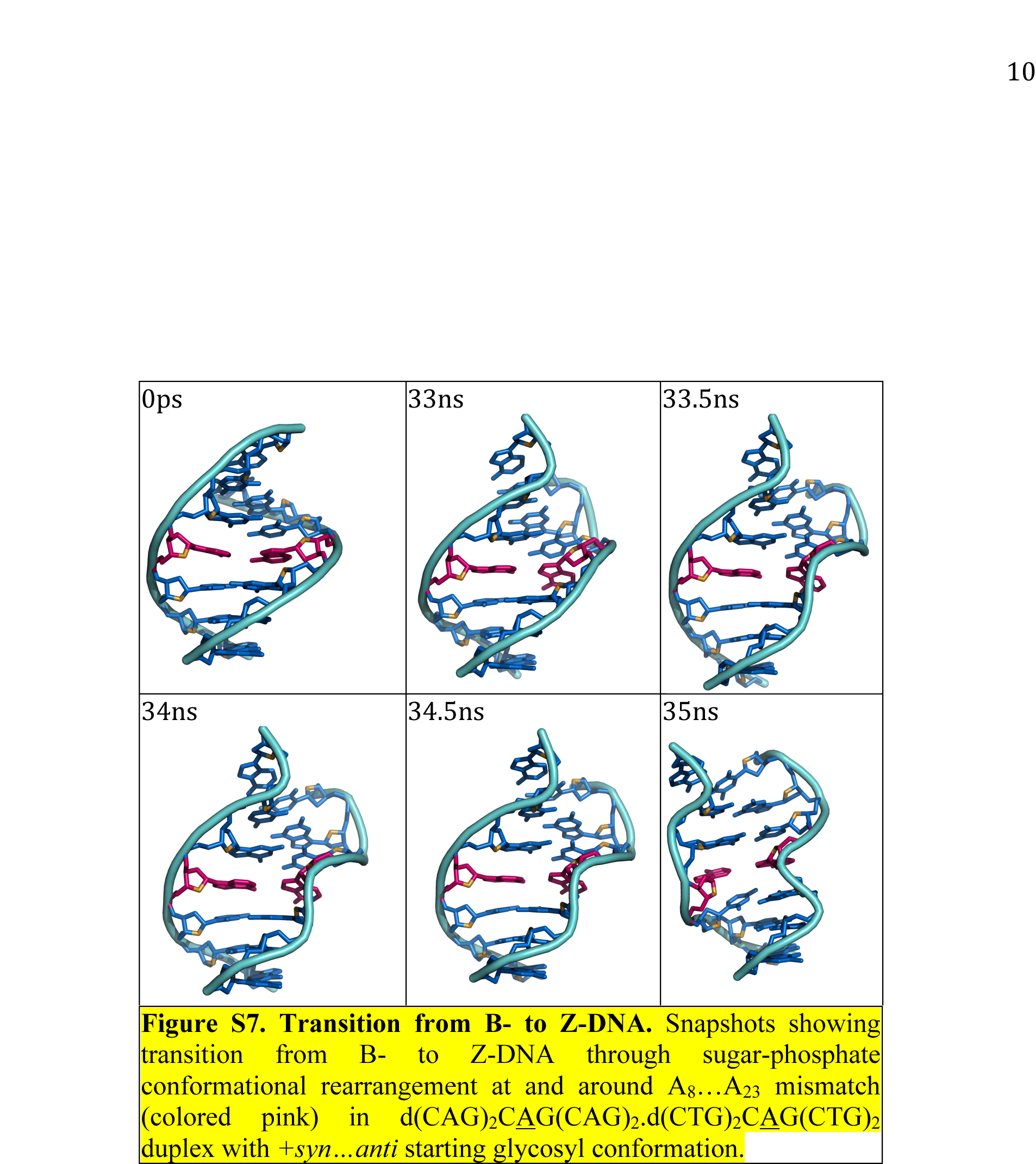

Supplement: S7 Fig — Snapshots showing transition from B- to Z-DNA through sugar-phosphate conformational rearrangement at and around A8…A23 mismatch (colored pink) in d(CAG)2CAG(CAG)2.d(CTG)2CAG(CTG)2 duplex with +syn…anti starting glycosyl conformation. (TIF) [file pcbi.1004162.s013.tif]

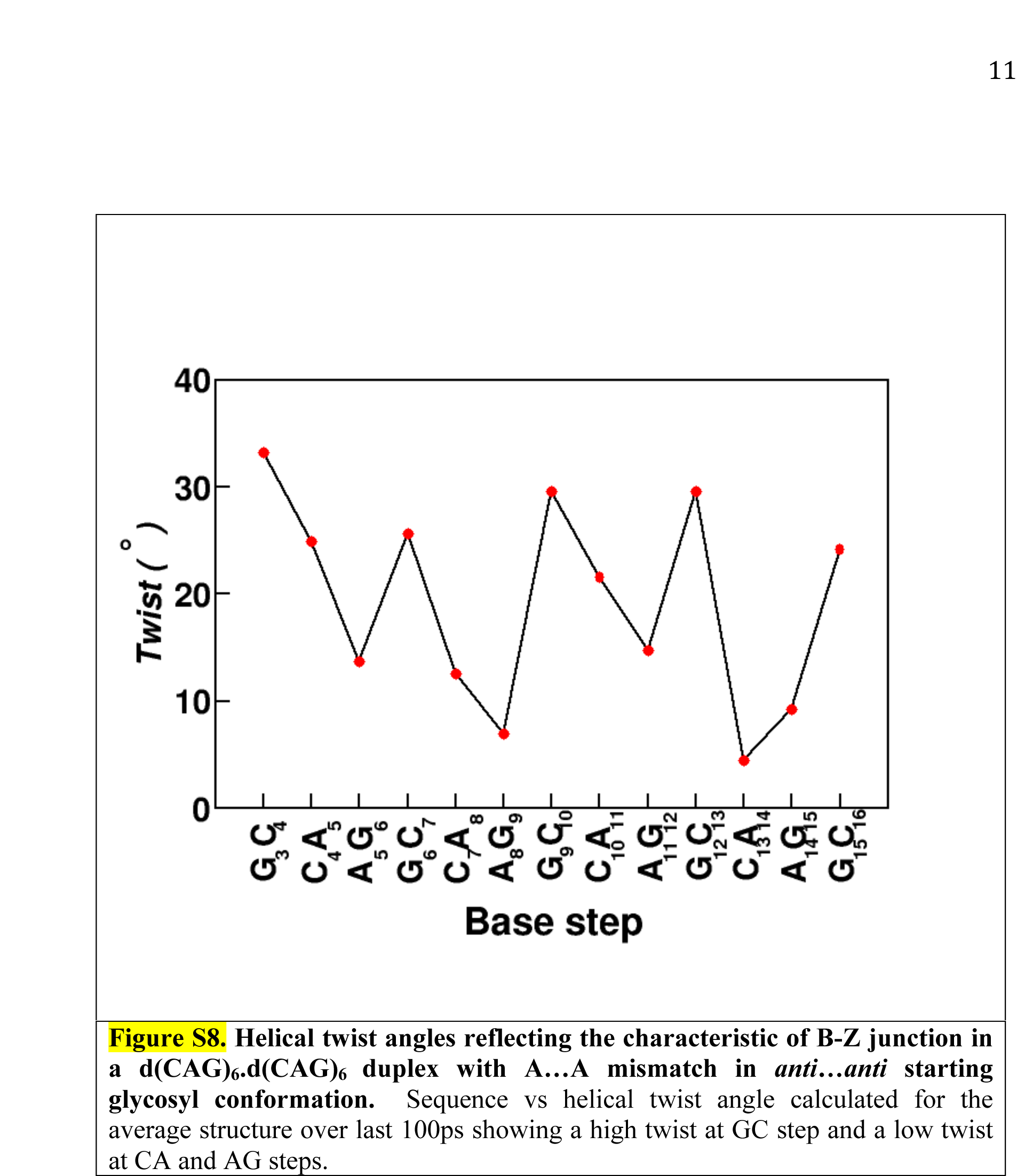

Supplement: S8 Fig — Sequence vs helical twist angle calculated for the average structure over last 100ps showing a high twist at GC step and a low twist at CA and AG steps. (TIF) [file pcbi.1004162.s014.tif]

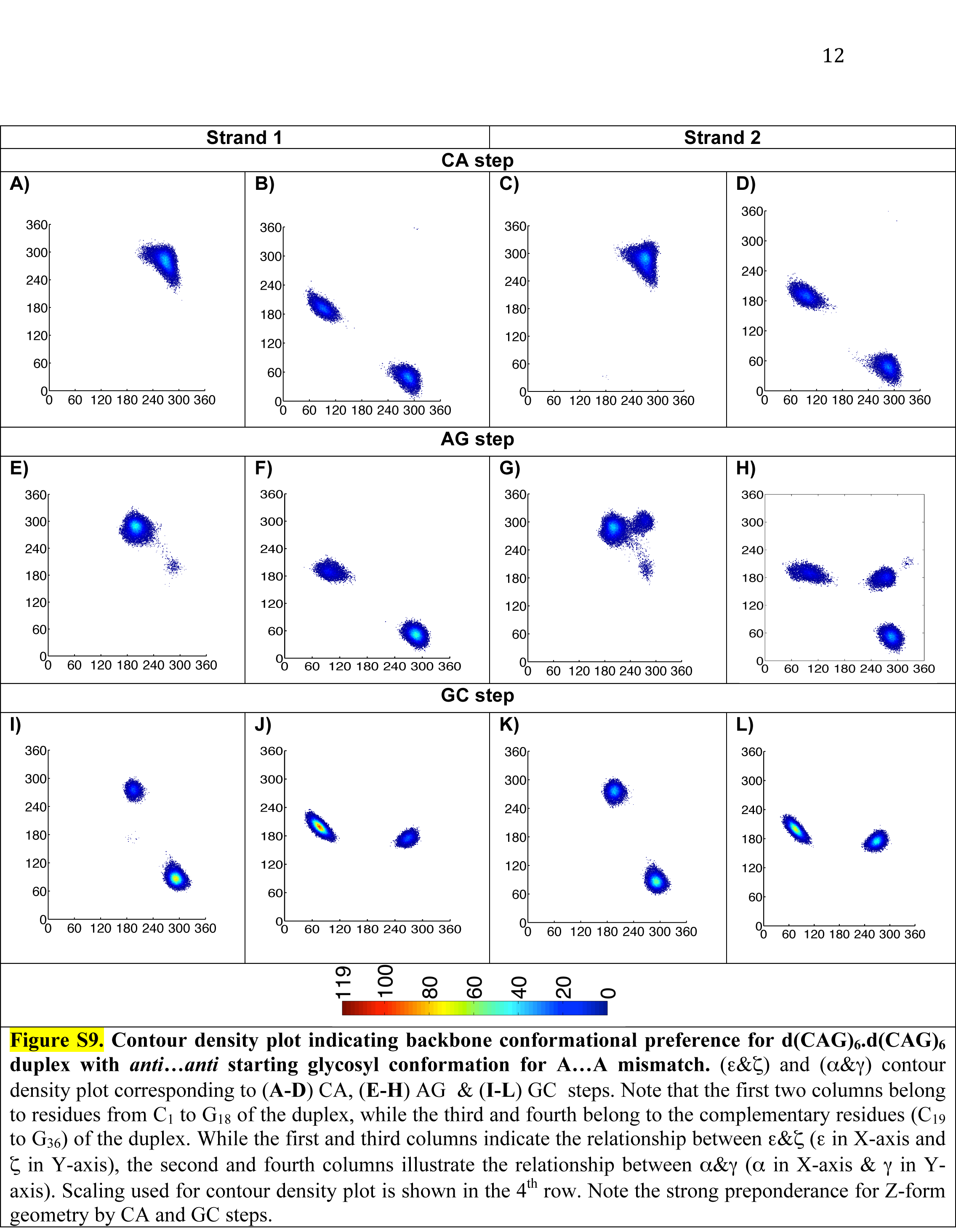

Supplement: S9 Fig — (ε&ζ) and (α&γ) contour density plot corresponding to (A-D) CA, (E-H) AG & (I-L) GC steps. Note that the first two columns belong to residues from C1 to G18 of the duplex, while the third and fourth belong to the complementary residues (C19 to G36) of the duplex. While the first and third columns indicate the relationship between ε & ζ (ε in X-axis and ζ in Y-axis), the second and fourth columns illustrate the relationship between α & γ (α in X-axis and γ in Y-axis). Scaling used for contour density plot is shown in the 4th row. Note the strong preponderance for Z-form geometry by CA and GC steps. (TIF) [file pcbi.1004162.s015.tif]

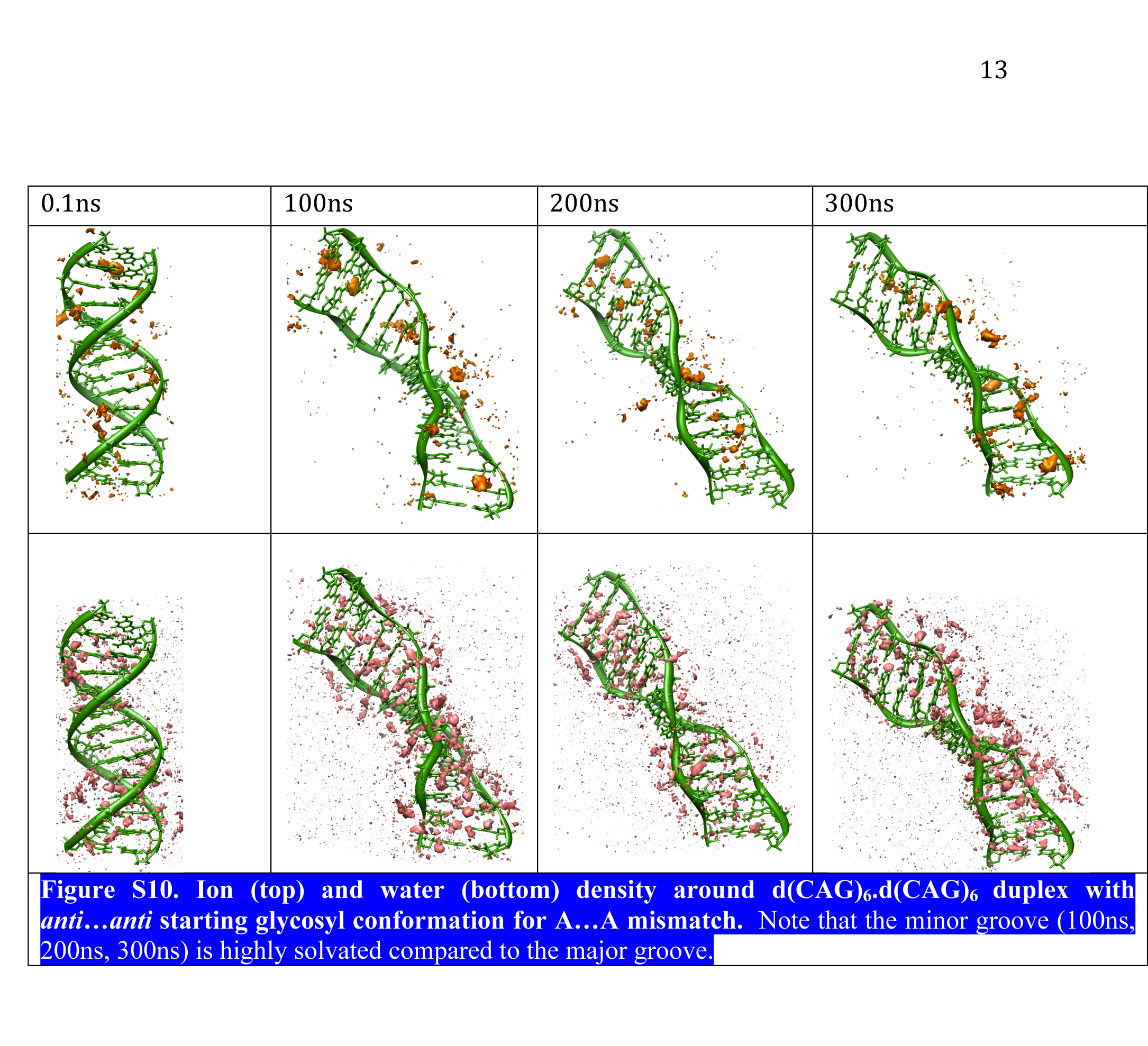

Supplement: S10 Fig — Note that the minor groove (100ns, 200ns, 300ns) is highly solvated compared to the major groove. (TIF) [file pcbi.1004162.s016.tif]

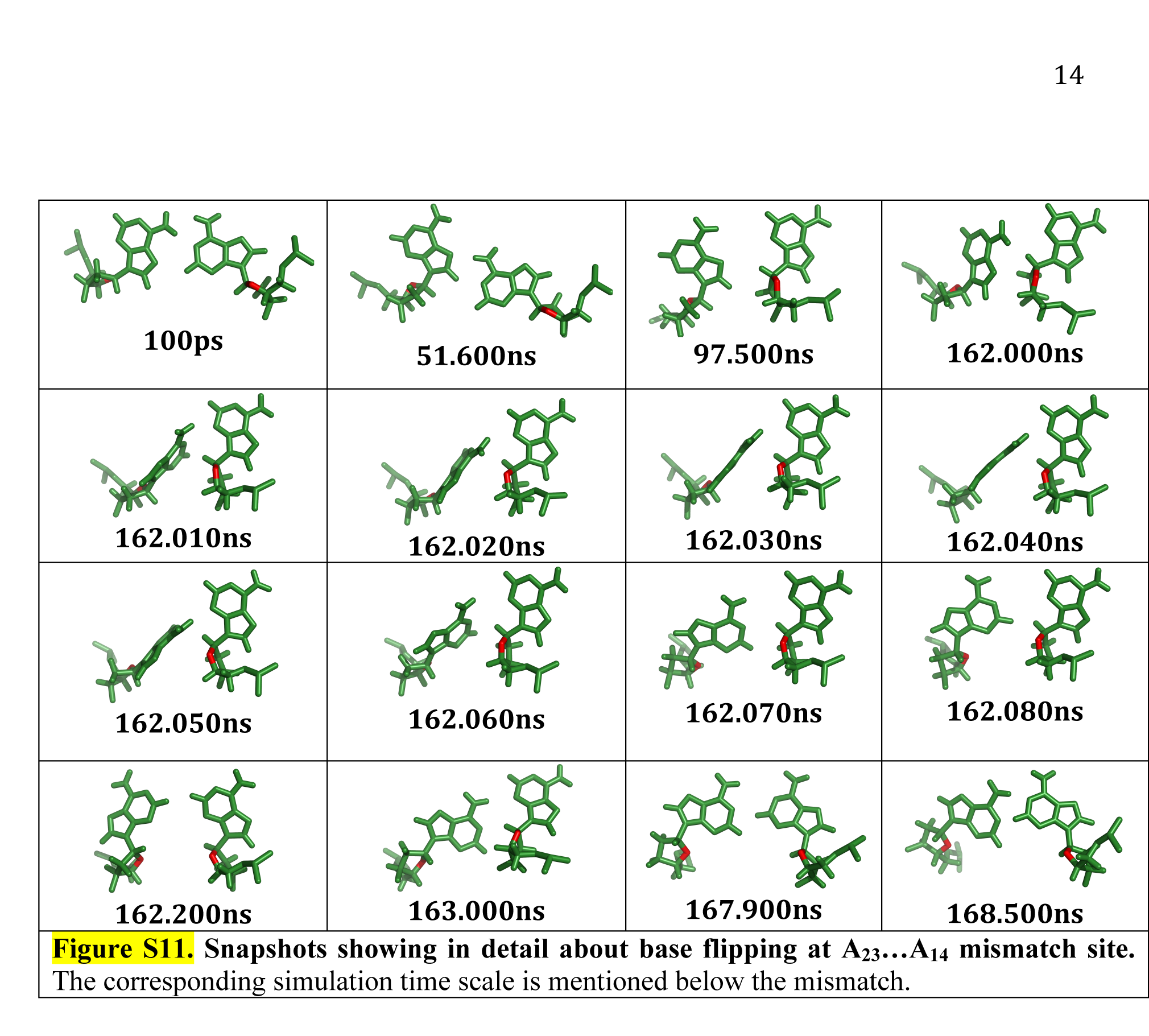

Supplement: S11 Fig — The corresponding simulation time scale is mentioned below the mismatch. (TIF) [file pcbi.1004162.s017.tif]

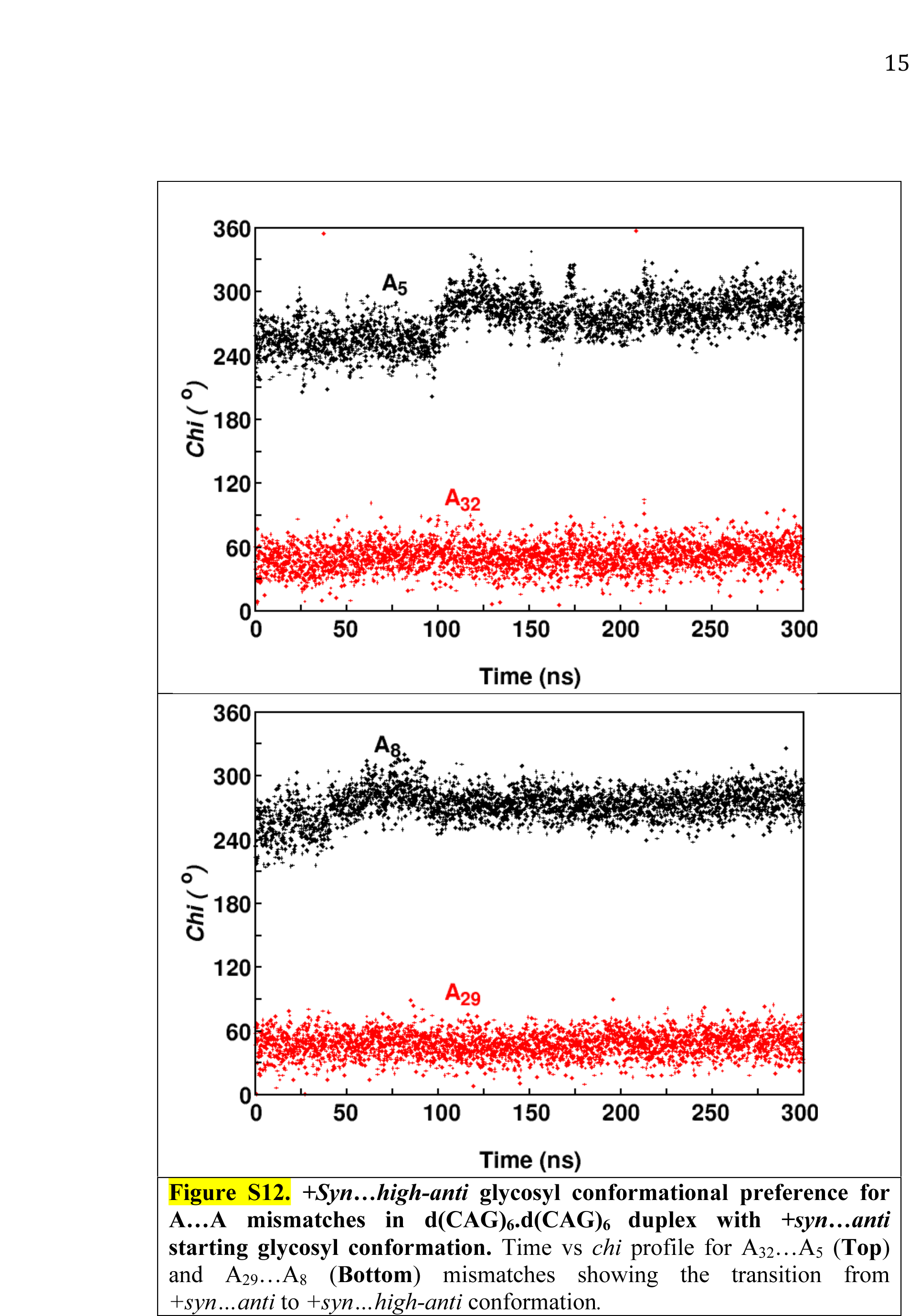

Supplement: S12 Fig — Time vs chi profile for A32…A5 (Top) and A29…A8 (Bottom) mismatches showing the transition from +syn…anti to +syn…high-anti conformation. (TIF) [file pcbi.1004162.s018.tif]

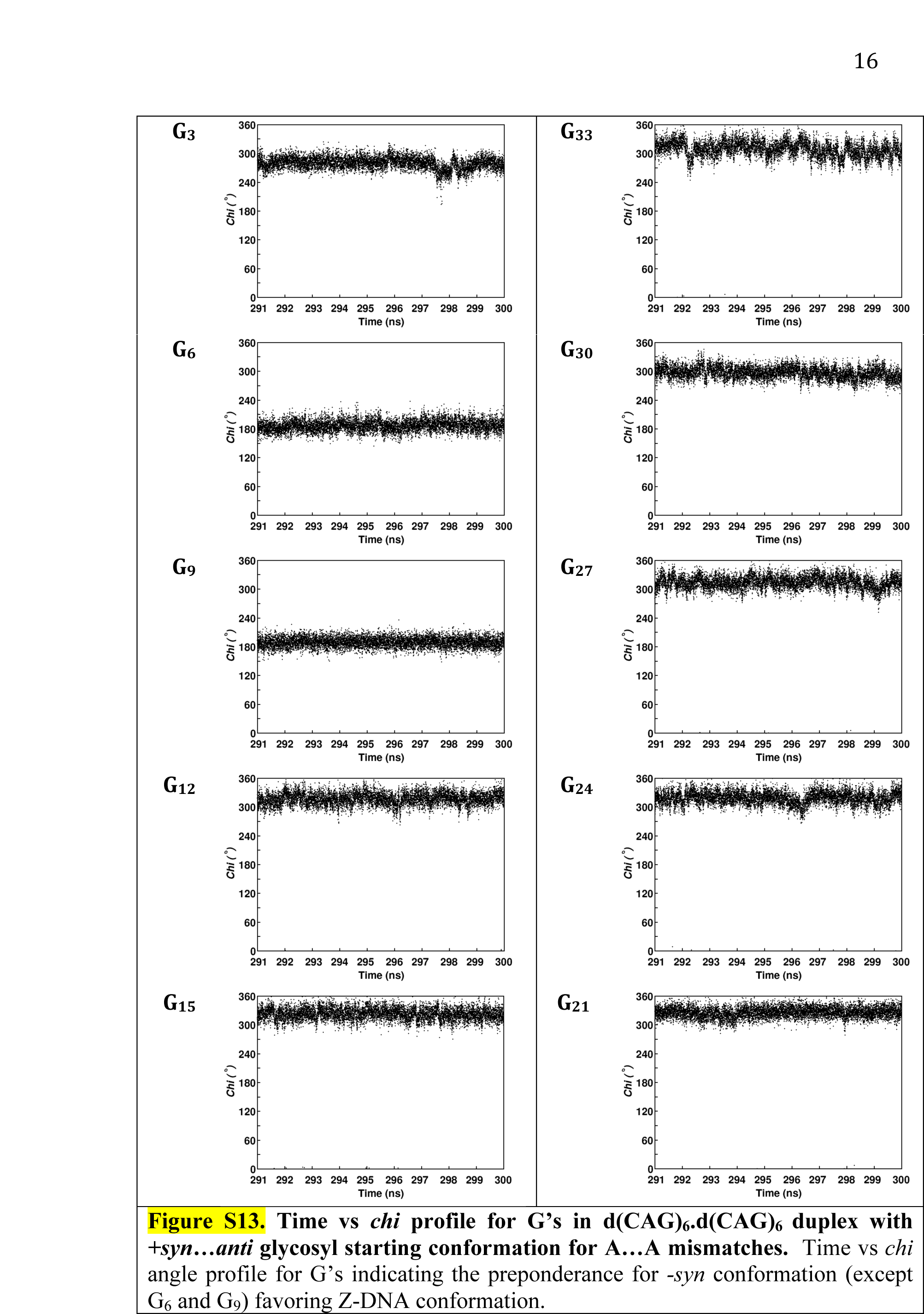

Supplement: S13 Fig — Time vs chi angle profile for G’s indicating the preponderance for -syn conformation (except G6 and G9) favoring Z-DNA conformation. (TIF) [file pcbi.1004162.s019.tif]

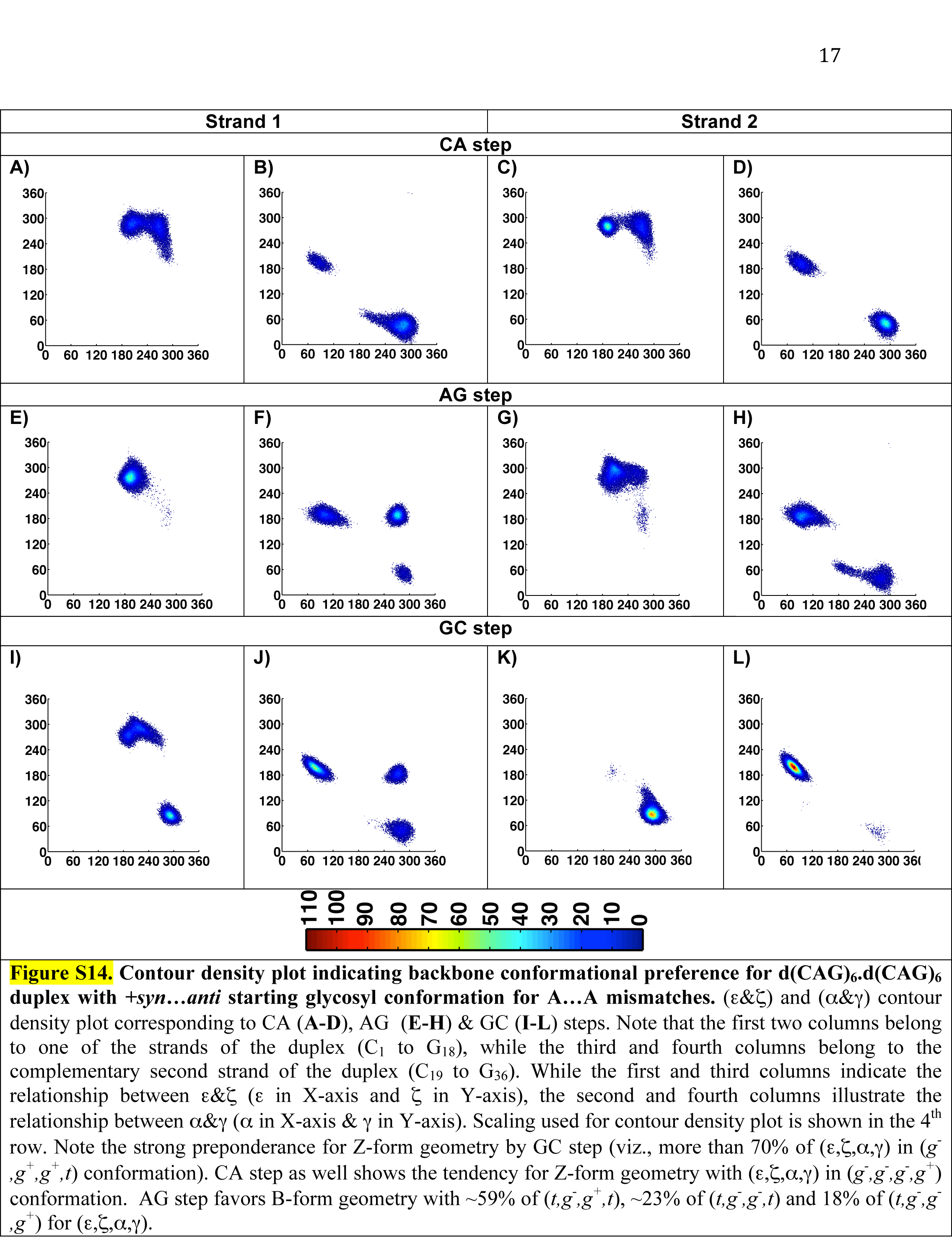

Supplement: S14 Fig — (ε&ζ) and (α&γ) contour density plot corresponding to CA (A-D), AG (E-H) & GC (I-L) steps. Note that the first two columns belong to one of the strands of the duplex (C1 to G18), while the third and fourth columns belong to the complementary second strand of the duplex (C19 to G36). While the first and third columns indicate the relationship between ε & ζ (ε in X-axis and ζ in Y-axis), the second and fourth columns illustrate the relationship between α & γ (α in X-axis and γ in Y-axis). Scaling used for contour density plot is shown in the 4th row. Note the strong preponderance for Z-form geometry by GC step (viz., more than 70% of (ε,ξ,α,γ) in (g -,g +,g +,t) conformation). CA step as well shows the tendency for Z-form geometry with (ε,ξ,α,γ) in (g -,g -,g -,g +) conformation. AG step favors B-form geometry with ~59% of (t,g -,g +,t), ~23% of (t,g -,g -,t) and 18% of (t,g -,g -,g +) for (ε,ξ,α,γ). (TIF) [file pcbi.1004162.s020.tif]

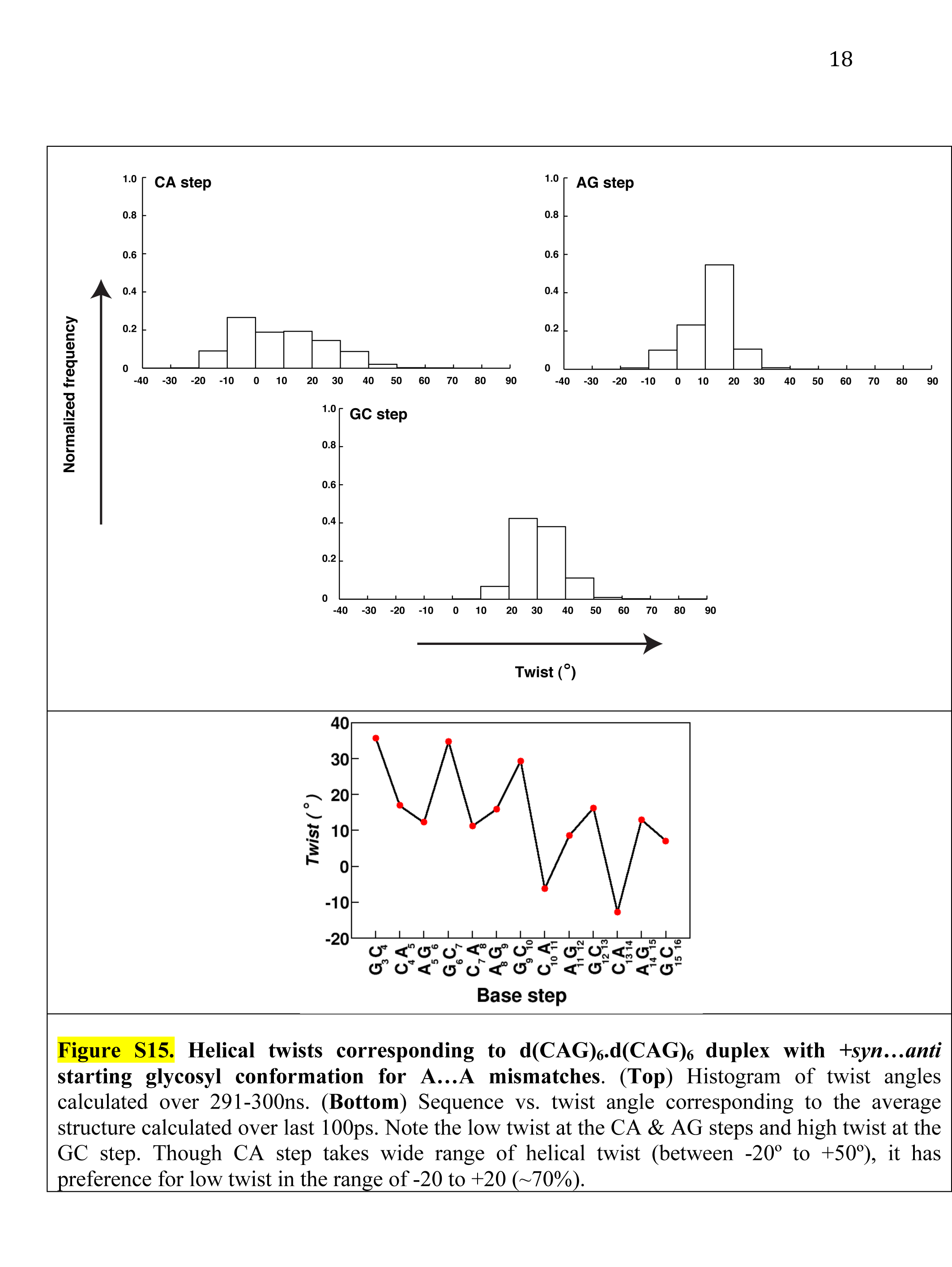

Supplement: S15 Fig — (Top) Histogram of twist angles calculated over 291-300ns. (Bottom) Sequence vs. twist angle corresponding to the average structure calculated over last 100ps. Note the low twist at the CA & AG steps and high twist at the GC step. Though CA step takes wide range of helical twist (between -20º to +50º), it has preference for low twist in the range of -20 to +20 (~70%). (TIF) [file pcbi.1004162.s021.tif]

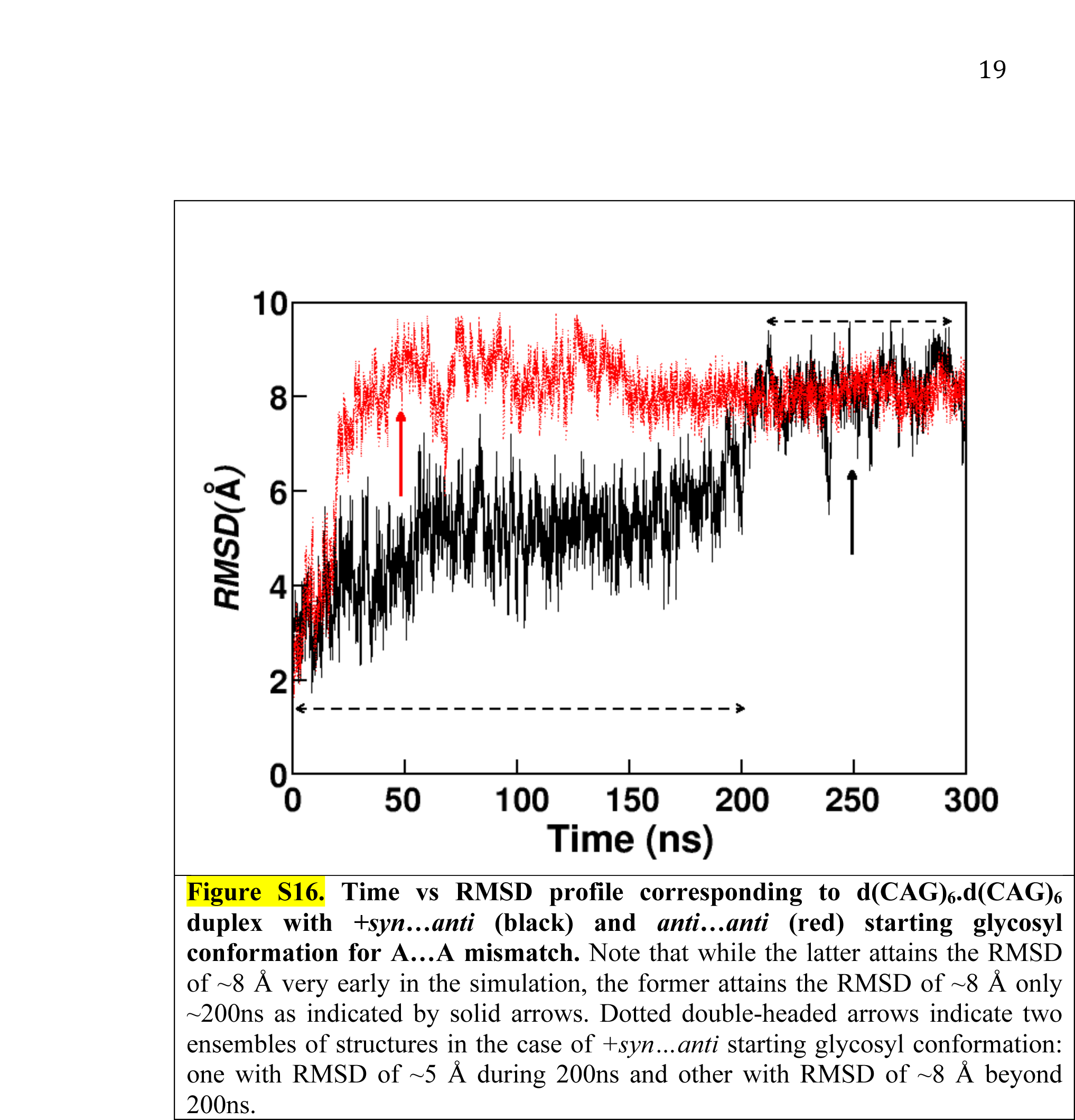

Supplement: S16 Fig — Note that while the latter attains the RMSD of ~8 Å very early in the simulation, the former attains the RMSD of ~8 Å only ~200ns as indicated by solid arrows. Dotted double-headed arrows indicate two ensembles of structures in the case of +syn…anti starting glycosyl conformation: one with RMSD of ~5 Å during 200ns and other with RMSD of ~8 Å beyond 200ns. (TIF) [file pcbi.1004162.s022.tif]

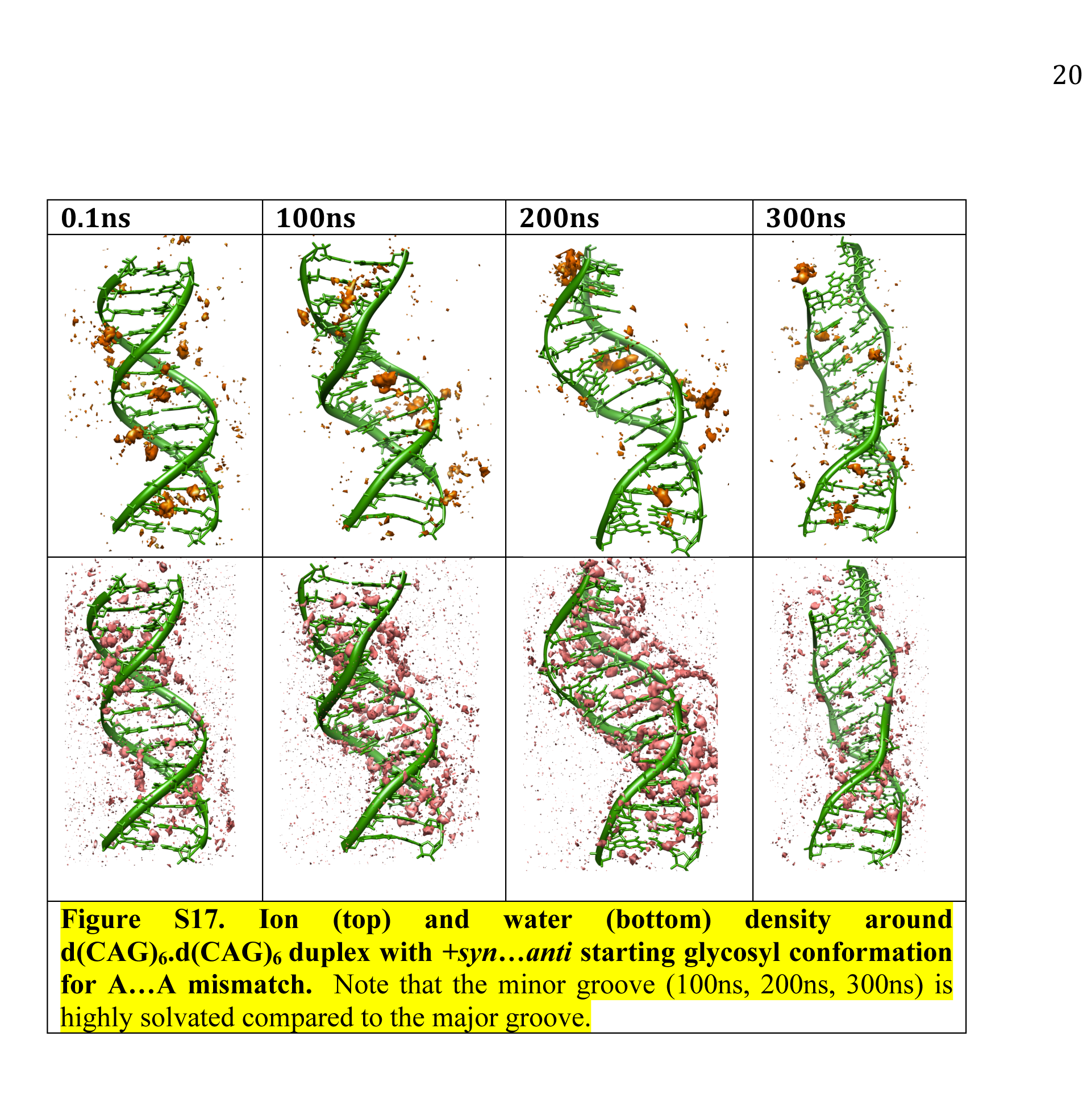

Supplement: S17 Fig — Note that the minor groove (100ns, 200ns, 300ns) is highly solvated compared to the major groove. (TIF) [file pcbi.1004162.s023.tif]

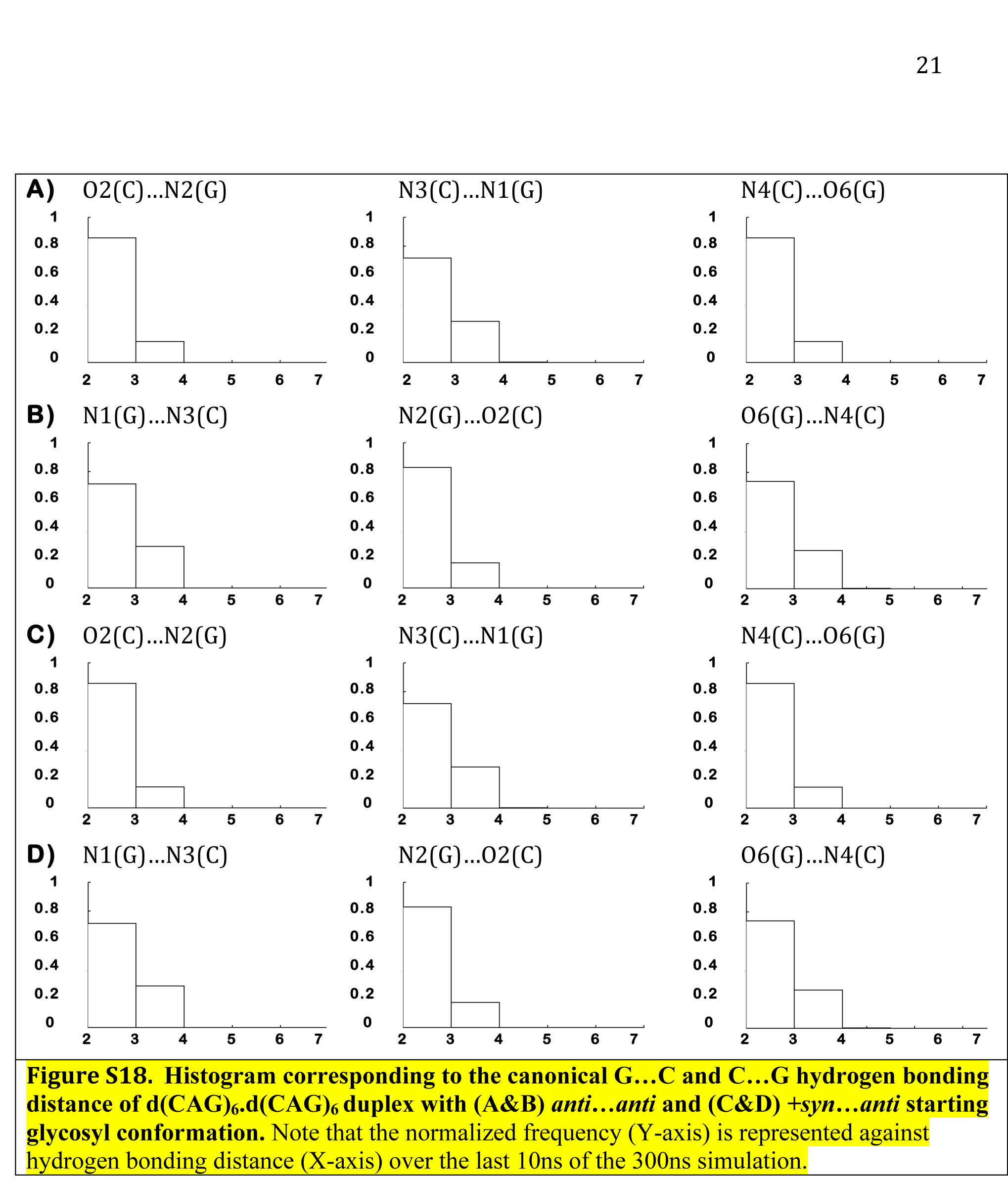

Supplement: S18 Fig — Note that the normalized frequency (Y-axis) is represented against hydrogen bonding distance (X-axis) over the last 10ns of the 300ns simulation. (TIF) [file pcbi.1004162.s024.tif]

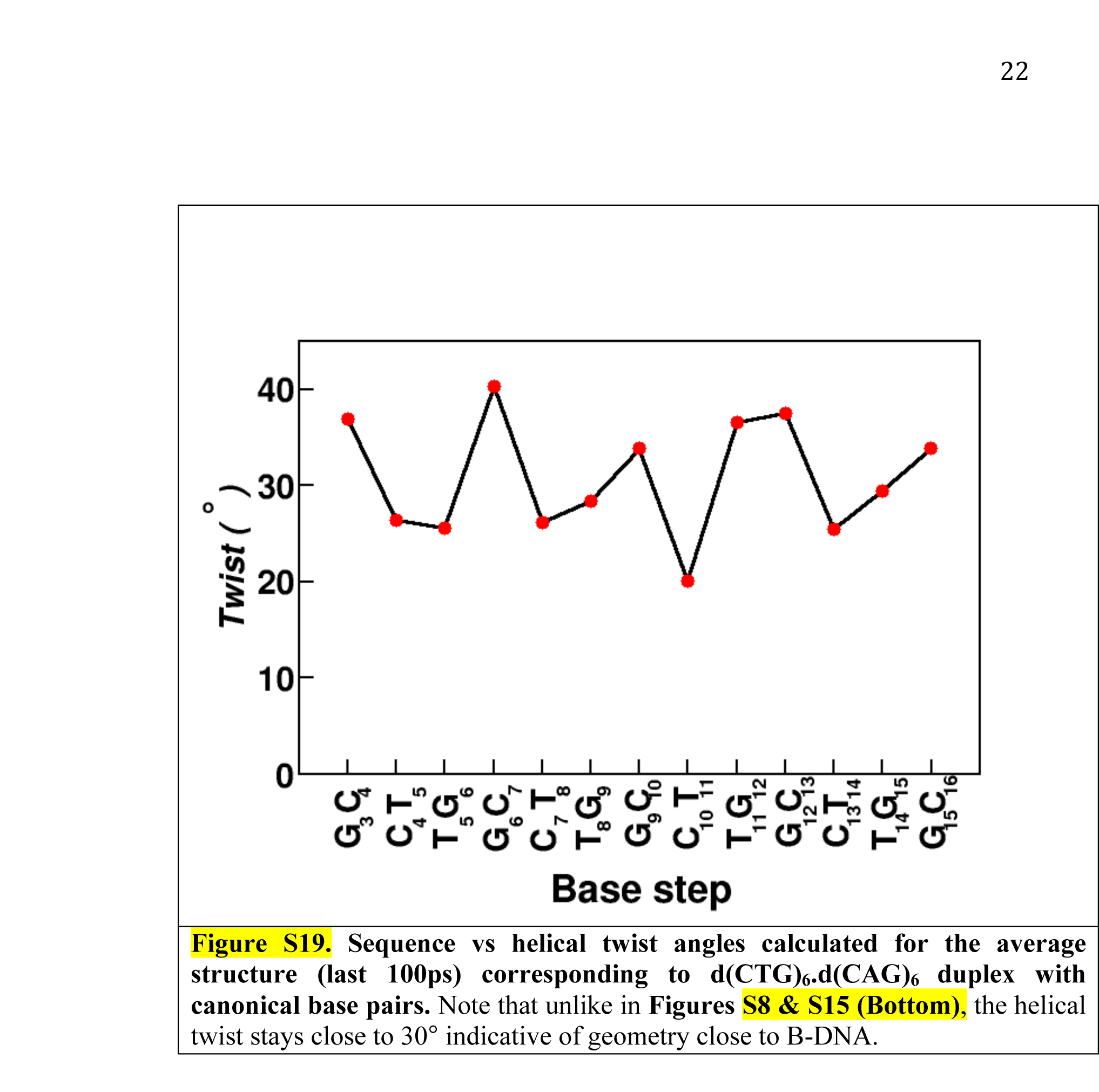

Supplement: S19 Fig — Note that unlike in S8&S15 (Bottom) Figs, the helical twist stays close to 30° indicative of geometry close to B-DNA. (TIF) [file pcbi.1004162.s025.tif]

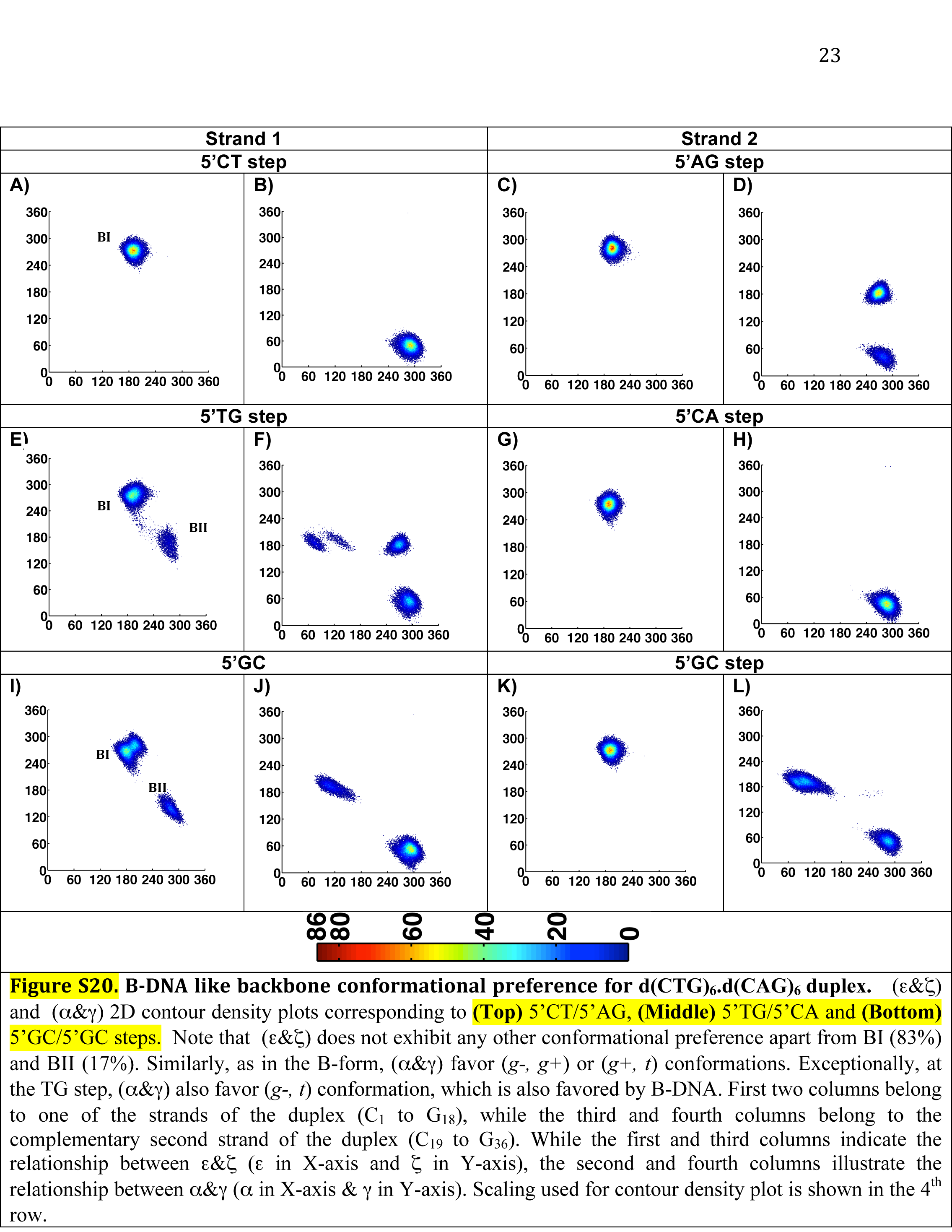

Supplement: S20 Fig — (ε&ζ) and (α&γ) 2D contour density plots corresponding to (Top) 5’CT/5’AG, (Middle) 5’TG/5’CA and (Bottom) 5’GC/5’GC steps. Note that (ε&ζ) does not exhibit any other conformational preference apart from BI (83%) and BII (17%). Similarly, as in the B-form, (α&γ) favor (g-, g+) or (g+, t) conformations. Exceptionally, at the TG step, (α&γ) also favor (g-, t) conformation, which is also favored by B-DNA. First two columns belong to one of the strands of the duplex (C1 to G18), while the third and fourth columns belong to the complementary second strand of the duplex (C19 to G36). While the first and third columns indicate the relationship between ε & ζ (ε in X-axis and ζ in Y-axis), the second and fourth columns illustrate the relationship between α & γ (α in X-axis and γ in Y-axis). Scaling used for contour density plot is shown in the 4th row. (TIF) [file pcbi.1004162.s026.tif]

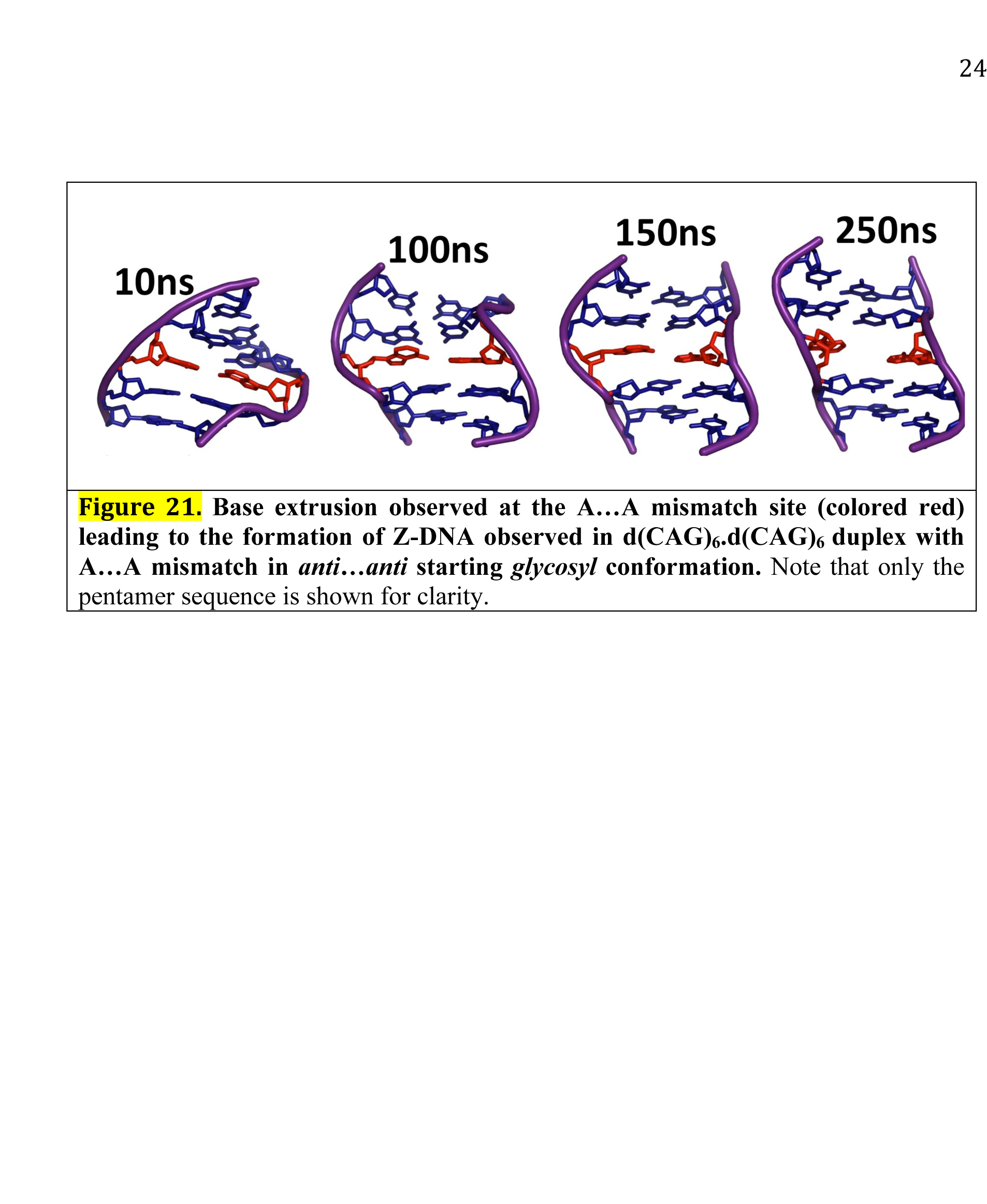

Supplement: S21 Fig — Note that only the pentamer sequence is shown for clarity. (TIF) [file pcbi.1004162.s027.tif]

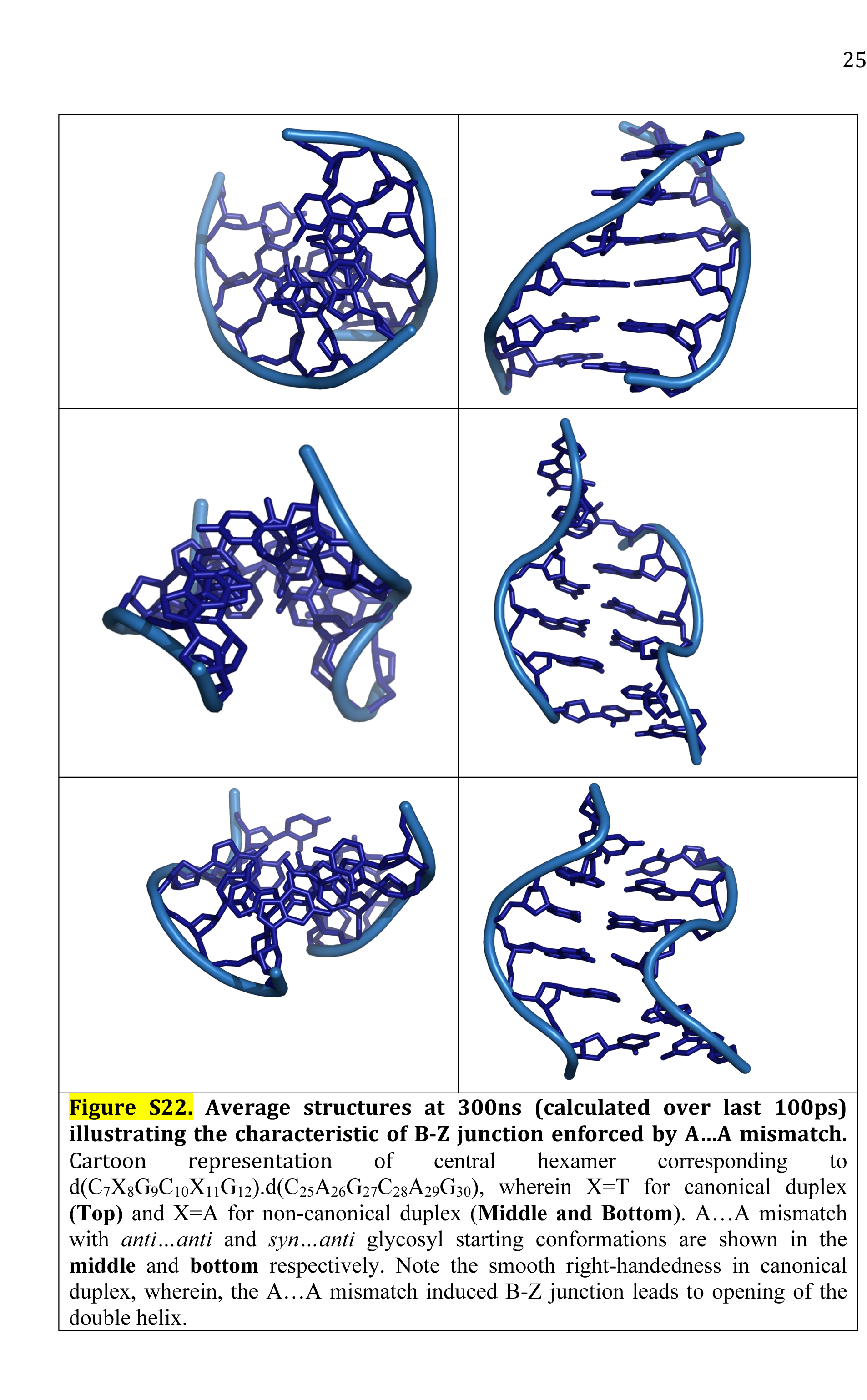

Supplement: S22 Fig — Cartoon representation of central hexamer corresponding to d(C7X8G9C10X11G12).d(C25A26G27C28A29G30), wherein X = T for canonical duplex (Top) and X = A for non-canonical duplex (Middle and Bottom). A…A mismatch with anti…anti and syn…anti glycosyl starting conformations are shown in the middle and bottom respectively. Note the smooth right-handedness in canonical duplex, whereas, the A…A mismatch induced B-Z junction leads to opening of the double helix. (TIF) [file pcbi.1004162.s028.tif]

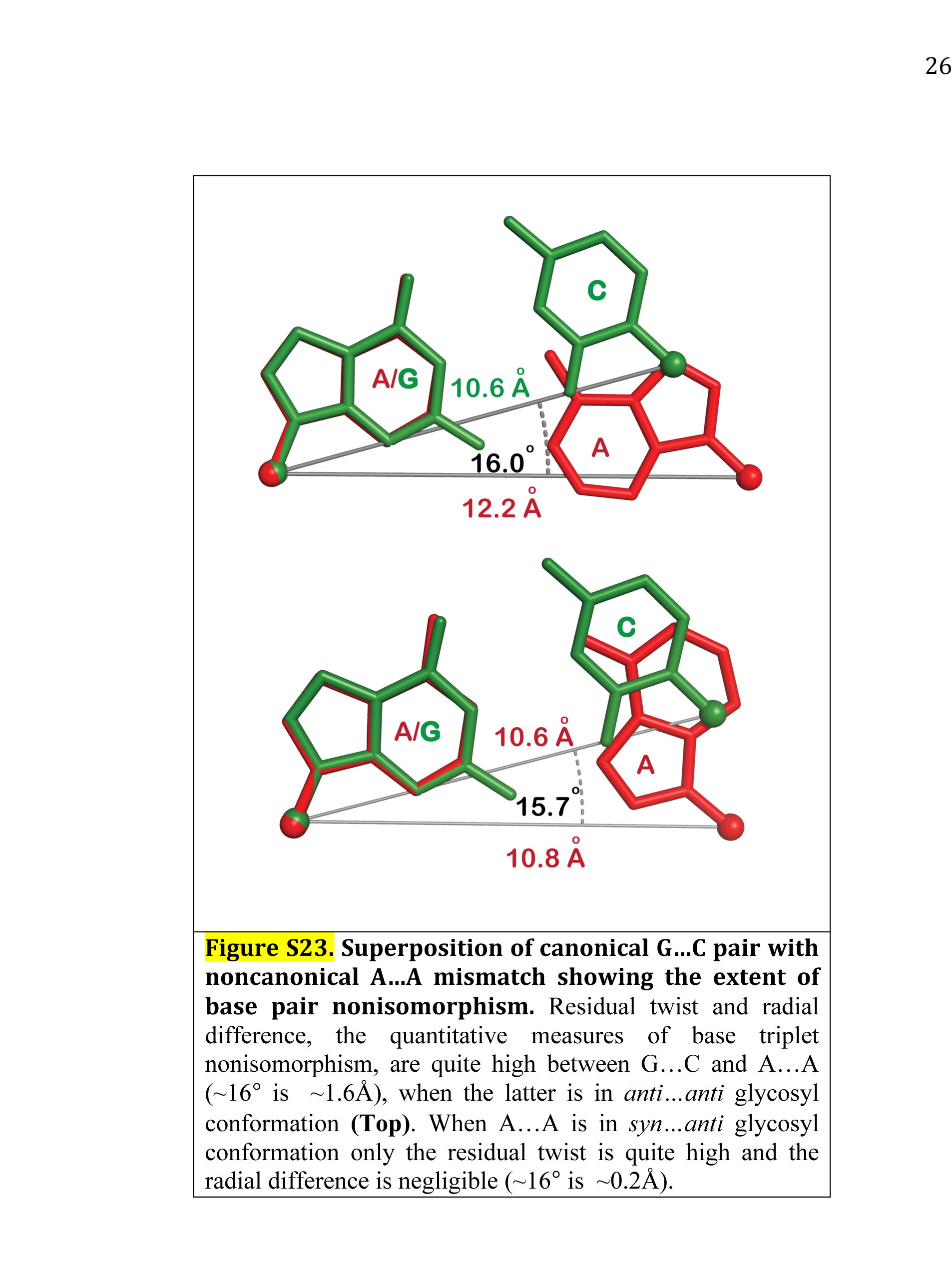

Supplement: S23 Fig — Residual twist and radial difference, the quantitative measures of base triplet nonisomorphism, are quite high between G…C and A…A (~16 is ~1.6Å), when the latter is in anti…anti glycosyl conformation (Top). When A…A is in syn…anti glycosyl conformation only the residual twist is quite high and the radial difference is negligible (~16 is ~0.2Å). (TIF) [file pcbi.1004162.s029.tif]
